# Mutated lncRNA increase the risk of type 2 diabetes by promoting β cell dysfunction and insulin resistance

**DOI:** 10.1038/s41419-022-05348-w

**Published:** 2022-10-27

**Authors:** Wan-Hui Guo, Qi Guo, Ya-Lin Liu, Dan-Dan Yan, Li Jin, Rong Zhang, Jing Yan, Xiang-Hang Luo, Mi Yang

**Affiliations:** 1 Department of Endocrinology, Endocrinology Research Center, Xiangya Hospital of Central South University, 410008 Changsha, Hunan P.R. China; 2 National Clinical Research Center for Geriatric Disorders, Xiangya Hospital, 410008 Changsha, Hunan P.R. China; 3 Shanghai Diabetes Institute, Shanghai Key Laboratory of Diabetes Mellitus, Shanghai Clinical Centre for Diabetes, Shanghai Sixth People’s Hospital affiliated to Shanghai Jiao Tong University School of Medicine, 200233 Shanghai, P.R. China

**Keywords:** Mechanisms of disease, Long non-coding RNAs, Type 2 diabetes, Type 2 diabetes, Type 2 diabetes

## Abstract

Islet β cell dysfunction and insulin resistance are the main pathogenesis of type 2 diabetes (T2D), but the mechanism remains unclear. Here we identify a rs3819316 C > T mutation in lncRNA *Reg1cp* mainly expressed in islets associated with an increased risk of T2D. Analyses in 16,113 Chinese adults reveal that *Mut-Reg1cp* individuals had higher incidence of T2D and presented impaired insulin secretion as well as increased insulin resistance. Mice with islet β cell specific *Mut-Reg1cp* knock-in have more severe β cell dysfunction and insulin resistance. Mass spectrometry assay of proteins after RNA pulldown demonstrate that *Mut-Reg1cp* directly binds to polypyrimidine tract binding protein 1 (PTBP1), further immunofluorescence staining, western blot analysis, qPCR analysis and glucose stimulated insulin secretion test reveal that *Mut-Reg1cp* disrupts the stabilization of insulin mRNA by inhibiting the phosphorylation of PTBP1 in β cells. Furthermore, islet derived exosomes transfer *Mut-Reg1cp* into peripheral tissue, which then promote insulin resistance by inhibiting AdipoR1 translation and adiponectin signaling. Our findings identify a novel mutation in lncRNA involved in the pathogenesis of T2D, and reveal a new mechanism for the development of T2D.

## Introduction

Diabetes and its complications are widespread in the world and have become one of the major threats to human health. According to data released by the International Diabetes Federation (IDF) in 2021 [1], about 537 million people worldwide are living with diabetes. Without sufficient action to address the pandemic, the number of people suffered from diabetes would raise to 783 million by 2045 [1, 2]. More than 90% of all diabetics have T2D, which is characterized by a decrease in the number of β cells or a decline of β cells function, resulting in an inability to compensate for the high insulin requirement in insulin resistant states [3, 4]. Islet β cell dysfunction and insulin resistance are the main pathogenesis of T2D, while the mechanism of which still remains complex and unclear.

Over the years, multiple genome-wide association studies have linked single nucleotide polymorphisms (SNPs) at more than 250 loci to T2D risk in the human genome, and the majority of diabetes susceptibility loci are mapped to non-coding regions [5–8]. Long non-coding RNAs (lncRNAs) are classified as non-protein coding transcripts longer than 200 nucleotides [9, 10], which regulate a wide range of biological processes such as cellular differentiation, proliferation, apoptosis, gene regulation, and cancer development via binding to DNA, RNA, or protein complexes [11–14]. lncRNAs have been shown to be involved in controlling β-cell proliferation, compensation, apoptosis and function [15, 16]. Understanding the functions of lncRNAs in β-cell biology might provide crucial insights into the pathogenesis of diabetes.

In our previous study, we identified a novel rs3819316 C > T mutation in lncRNA -*Reg1cp*, which is associated with high bone mass (HBM) by directly binding to Krüppel-like factor 3 to regulate angiogenesis [17]. It’s worth noting that *Reg1cp* belongs to Reg family and is a pseudogene which is conservedly expressed in human and is mainly expressed in pancreas, especially in islet [18, 19]. So, we further investigate the role of *Reg1cp* in glucose homeostasis. In the present study, we demonstrate this mutation in *Reg1cp* promotes T2D development both by impaired islet β cell function and increased insulin resistance.

## Results

### rs3819316 C > T mutation in *Reg1cp* gene is a risk factor of type 2 diabetes

In our previous study we identified a rs3819316 C > T mutation in *Reg1cp* gene which is associated with HBM [17]. As *Reg1cp* is mainly expressed in pancreas, especially in islet [18, 19], we further analyzed glucose metabolism related indexes in Chinese population (*n* = 16,113, including 6,744 males and 9,369 females). Among them, 1554 heterozygous *Reg1cp* individuals (*Reg1cp*^*+/mut*^) and 90 homozygote *Reg1cp* individuals (*Reg1cp*^*mut/mut*^) were identified. These *Reg1cp*^*+/mut*^ and *Reg1cp*^*mut/mut*^ individuals had higher incidence of diabetes and prediabetic states compared with those with the *WT* gene (Table 1). Significant correlations of rs3819316 C > T mutation with diabetes and prediabetic states were observed after adjusting for gender, age and body mass index (BMI) (OR 1.44 [95% CI 1.29–1.61], *p* = 9.52e−11; OR 1.39 [95% CI 1.24–1.57], *p* = 6.57e–08, respectively).

**Table 1:** Clinical characteristics of the total population.

	TT	TC	CC	*p* value
N	90	1,554	14,469	–
NGT	23	481	6,634	<0.0001
Pre-diabetes	37	618	5,092	
T2DM	30	455	2,743	
Gender (male/female)	36/54	691/863	6017/8452	0.0858
Age (year)	54.9 ± 10.0	58.3 ± 7.9	54 ± 11.1	<0.0001
BMI (kg/m^2^)	25.4 ± 5.1	25.1 ± 3.5	24.6 ± 3.3	<0.0001
HbA1c (%)	6.0 (5.5, 6.4)	5.7 (5.4, 6.3)	5.7 (5.4, 6.1)	<0.0001
FPG (mmol/L)	5.8 (5.4, 6.8)	6.0 (5.5, 6.7)	5.7 (5.1, 6.3)	<0.0001
PG30 (mmol/L)	9.6 (8.8, 11.5)	10.3 (9.3, 11.8)	9.4 (8.3, 11.1)	<0.0001
PG2H (mmol/L)	8.3 (6.7, 12.6)	8.5 (6.8, 11.3)	7.5 (5.8, 9.5)	<0.0001

Data are presented as mean ± standard deviation or median (interquartile range). N. Kruskal Wallis test for continuous variables and χ^2^ test for categorical variables were applied to assess the differences among three groups.

*NGT* normal glucose tolerance, *T2DM* type 2 diabetes mellitus, *BMI* body mass index, *HbA1c* glycated hemoglobin A1c, *FPG* fasting plasma glucose, *PG30* 30 min plasma glucose, *PG2H* 2 h plasma glucose.

**Fig. 1:**
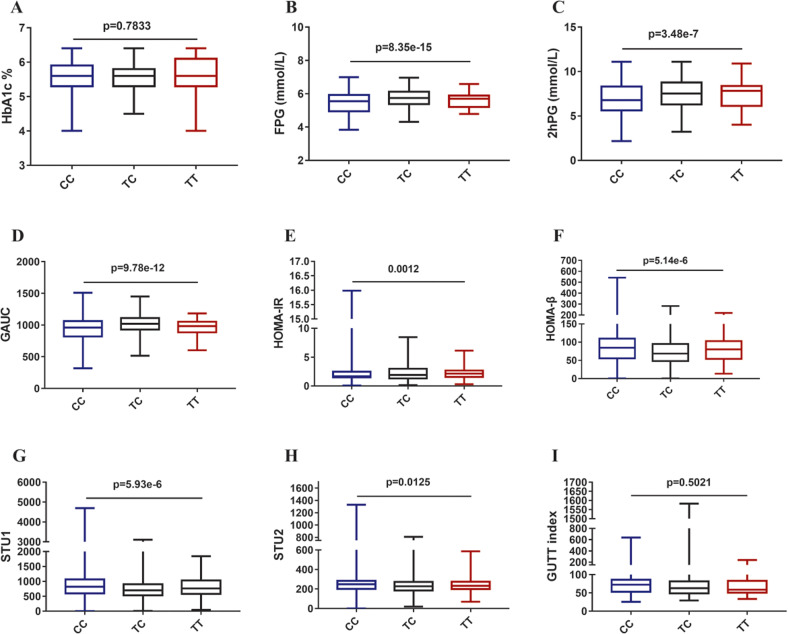
Associations between rs3819316 C > T mutation and glucose metabolism related indexes. Associations of rs3819316 C > T mutation with glucose metabolism related indexes were analyzed after adjustment for gender, age and body mass index (BMI). **A** Analysis of glycated hemoglobin A1c (HbA1c); **B** Analysis of fasting plasma glucose (FPG); **C** Analysis of 2 h plasma glucose (2hPG) based on oral glucose tolerance test; **D** Analysis of the area under the curve of the glucose (GAUC) from 0 to 120 min based on oral glucose tolerance test; **E** Analysis of homeostasis model assessment of insulin resistance (HOMA-IR); **F** Analysis of homeostasis model assessment of β cell function (HOMA-β); **G** Analysis of Stumvoll first-phase insulin secretion (STU1); **H** Analysis of Stumvoll second-phase insulin secretion (STU2). **I** Analysis of GUTT index based on oral glucose tolerance test. Data shown as median (interquartile range), N. Kruskal Wallis test for continuous variables and χ2 test for categorical variables were applied to assess the differences among three groups.

To find more clues beneath the connection between rs3819316 C > T mutation and diabetes, further analyses for glucose metabolism related indexes were performed in 12,885 non-diabetic individuals, as shown in Fig. 1, the rs3819316 C > T mutation was significantly associated with fasting plasma glucose (FPG), 2 h plasma glucose (2hPG), glucose area under curve (GAUC), homeostasis model assessment of insulin resistance (HOMA-IR), homeostasis model assessment of β cell function (HOMA-β), Stumvoll first phase insulin secretion (STU1), and Stumvoll second phase insulin secretion (STU2). These results suggested that *Reg1cp* might be a new diabetes associated gene.

### Mutant *Reg1cp* impaired the glucose metabolism in mice

To investigate the impact of wild-type *Reg1cp* (*WT-Reg1cp*) and mutant *Reg1cp* (*Mut-Reg1cp*) on glucose metabolism in vivo, we constructed transgenic mice expressing *WT-Reg1cp* or *Mut-Reg1cp* in insulin positive pancreatic islet β cells (*Reg1cp-wt*_*RIP*_^*+*^ mice or *Reg1cp-mut*_*RIP*_^*+*^ mice) by intercrossing mice carrying Rosa26-loxP-flanked-stop cassette controlling *WT-Reg1cp* or *Mut-Reg1cp* alleles expression (*Rosa-Reg1cp-wt* mice or *Rosa-Reg1cp-mut* mice) with *RIP-Cre* transgenic mice that express Cre recombinase under the control of the rat insulin 2 promoter [20]. As expected, the expression of *Reg1cp* was detected in the islet of transgenic mice by qPCR and *Reg1cp* FISH with insulin immunofluorescent staining. Sanger sequencing further confirmed the successful knock-in of *WT-Reg1cp* or *Mut-Reg1cp* (Supplementary Fig. 1A–D). *Reg1cp-wt*_*RIP*_^*+*^ mice and *Reg1cp-mut*_*RIP*_^*+*^ mice had normal pancreatic exocrine elements including ducts and acini, and scattered endocrine islets with normal distribution (Supplementary Fig. 1E).

**Fig. 2:**
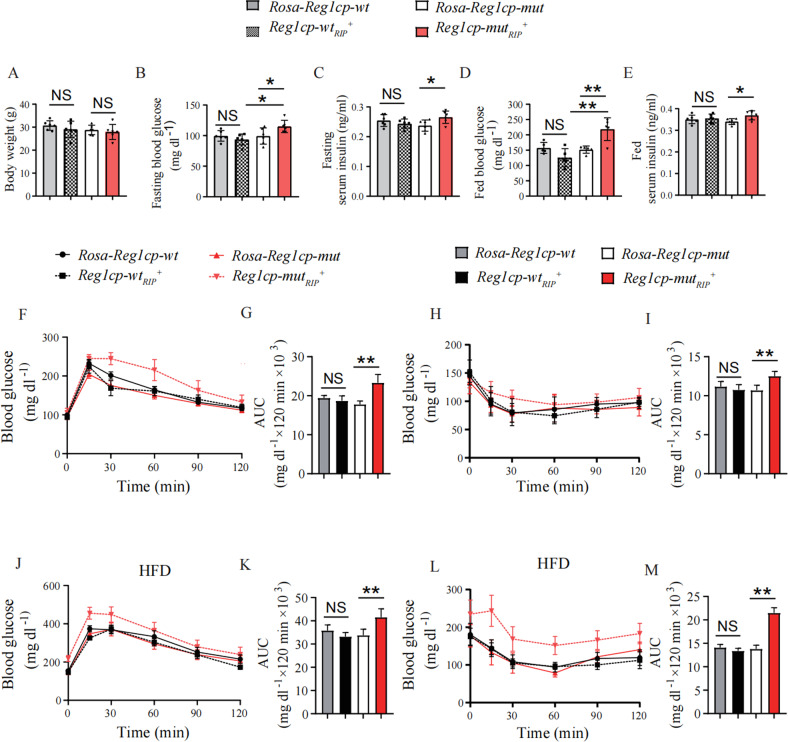
*Mut-Reg1cp* impaired the glucose metabolism in mice. **A**–**E** The body weight (**A**), fasting blood glucose level (**B**), fasting serum insulin level (**C**), fed blood glucose level (**D**) and fed serum insulin level (**E**) of *Rosa-Reg1cp-wt*, *Rosa-Reg1cp-mut*, *Reg1cp-wt*_*RIP*_^*+*^ and *Reg1cp-mut*_*RIP*_^*+*^ mice under regular diet. **F**–**I** The GTTs (**F**) and ITTs (**H**) in *Reg1cp-wt*_*RIP*_^*+*^, *Reg1cp-mut*_*RIP*_^*+*^ mice and related *Rosa-Reg1cp-wt* and *Rosa-Reg1cp-mut* controls under regular diet. Bar graphs showing the calculated values for area under curve (AUC) during the 120-min test course of the GTTs (**G**) and ITTs (**I**) (mg dl^−1^ × 120 min × 10^3^). **J**–**M** The GTTs (**J**) and ITTs (**L**) in *Reg1cp-wt*_*RIP*_^*+*^, *Reg1cp-mut*_*RIP*_^*+*^ mice and related *Rosa-Reg1cp-wt* and *Rosa-Reg1cp-mut* controls under HFD. Bar graphs showing the calculated values for area under curve (AUC) during the 120-min test course of the GTTs (**K**) and ITTs (**M**) (mg dl^−1^ × 120 min × 10^3^). *n* = 6 in each group from three independent experiments. Data shown as mean ± SD. *P < 0.05; **P < 0.01; NS no significance; One-way ANOVA.

When maintained on regular diet, *Reg1cp-wt*_*RIP*_^*+*^ mice showed no differences in body weight, fasting blood glucose and fasting serum insulin levels as compared with *Rosa-Reg1cp-wt* control mice (Fig. 2A–C). The fed blood glucose and fed serum insulin levels of *Reg1cp-wt*_*RIP*_^*+*^ mice also remained indistinguishable as compared with *Rosa-Reg1cp-wt* control mice when maintained on regular diet (Fig. 2D, E). Consistently, the glucose tolerance and clearance of *Reg1cp-wt*_*RIP*_^*+*^ mice also had no obvious changes compared with *Rosa-Reg1cp-wt* control mice, as shown by the results of glucose tolerance tests (GTTs) (Fig. 2F, G) and insulin tolerance tests (ITTs) (Fig. 2H, I). These results indicated that *WT-Reg1cp* knock-in did not affect the glucose metabolism in mice under regular diet. However, the fasting and fed blood glucose and serum insulin levels were increased in *Reg1cp-mut*_*RIP*_^*+*^ *mice* as compared with *Rosa-Reg1cp-mut* littermate controls (Fig. 2B–E). The results of GTTs and ITTs indicated the glucose tolerance and clearance of *Reg1cp-mut*_*RIP*_^*+*^ mice were worse than *Rosa-Reg1cp-mut* controls (Fig. 2F–I). In general, these results demonstrated that *Mut-Reg1cp* affected glucose homeostasis in mice.

Metabolic stressors, such as high-fat diet (HFD) could accelerate the disruption of glucose homeostasis. We next examined animals maintained on HFD. After 12-week HFD started at 12 weeks of age, we found that the impaired glucose tolerance as judged by GTTs and deteriorated insulin resistance as detected by ITTs induced by HFD were aggravated in *Reg1cp-mut*_*RIP*_^*+*^ mice compared with their littermate controls (Fig. 2J–M). The fasting blood glucose levels and fasting serum insulin levels were increased in *Reg1cp-mut*_*RIP*_^*+*^ *mice* compared with *Rosa-Reg1cp-mut* littermate controls when maintained on HFD (Supplementary Fig. 1G, H). However, the body weight, fasting blood glucose levels, fasting serum insulin levels, glucose tolerance and clearance showed no significant differences between *Reg1cp*-wt_RIP_^+^ mice and *Rosa-Reg1cp-wt* control mice (Fig. 2J–M, Supplementary Fig. 1F–H). Those results indicated that *WT-Reg1cp* did not affect glycometabolism and *Mut-Reg1cp* disrupted glucose homeostasis with or without metabolic stress.

### *Mut-Reg1cp* aggravated hyperglycemia by impaired β cell function

**Fig. 3:**
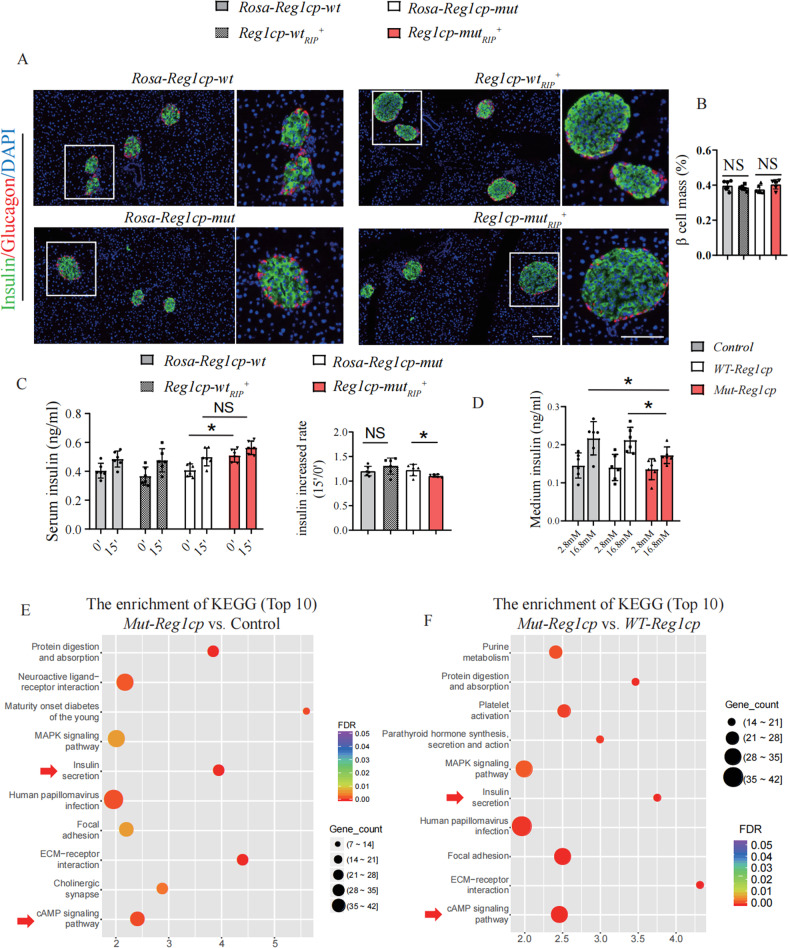
*Mut-Reg1cp* aggravated hyperglycemia by impaired β cell function. **A**, **B** Representative immunostaining images of insulin (green), glucagon (red) and DAPI, nucleus (blue) (**A**) and quantification of β cell mass (**B**) in *Reg1cp-wt*_*RIP*_^*+*^, *Reg1cp-mut*_*RIP*_^*+*^ mice and related *Rosa-Reg1cp-wt* and *Rosa-Reg1cp-mut* controls under regular diet. Scale bar: 100 μm. **C** In vivo GSIS test analysis of glucose stimulated insulin level (left panel) and the increased rate of insulin after glucose stimulation (right panel) of *Rosa-Reg1cp-wt*, *Rosa-Reg1cp-mut*, *Reg1cp-wt*_*RIP*_^*+*^ and *Reg1cp-mut*_*RIP*_^*+*^ mice (0’ and 15’ means 0 and 15 min after an intraperitoneal injection of glucose). **D** In vitro GSIS test analysis of glucose stimulated insulin level in MIN6 cells transfected with Control, *Mut-Reg1cp* or *WT-Reg1cp* plasmids. **E** Top 10 changed cellular functions which differentially expressed genes enriched in indicated by KEGG-enrichment analysis of MIN6 cells transfected with *Mut-Reg1cp* or Control plasmids. **F** Top 10 changed cellular functions which differentially expressed genes enriched in indicated by KEGG-enrichment analysis of MIN6 cells transfected with *Mut-Reg1cp* or *WT-Reg1cp* plasmids. In **A**–**D**, n = 6 in each group from three independent experiments. In **E**, **F**, *n* = 3. Data shown as mean ± SD. *P < 0.05; NS no significance; One-way ANOVA.

To further investigate the underlying mechanism of *Mut-Reg1cp* regulating glycometabolism, we evaluated the β cell mass, proliferation and senescence by immunofluorescent staining and analyzed the function of β cell by glucose stimulated insulin secretion (GSIS) test between these transgenic mice. The β cell mass of *Reg1cp-wt*_*RIP*_^*+*^ mice and *Reg1cp-mut*_*RIP*_^*+*^ mice remained indistinguishable as compared with their littermate controls whether maintained on regular diet (Fig. 3A, B) or HFD (Supplementary Fig. 2A, B). The Ki-67 positive or P21 positive β cells showed no significant differences between the transgenic mice and their littermate controls whether maintained on regular diet or HFD, indicating that neither *WT-* nor *Mut-Reg1cp* affected the proliferation and senescence of β cells (Supplementary Fig. 2C–H). SA-β Gal staining of MIN6 cells also showed no difference in senescence after transfection with *Mut-Reg1cp* or *WT-Reg1cp* plasmids (Supplementary Fig. 2I–J).

However, the β cell function was impaired in *Reg1cp-mut*_*RIP*_^*+*^ mice, as indicated by in vivo GSIS test (Fig. 3C). The impaired β cell function was also confirmed by in vitro GSIS test of MIN6 cells transfected with *Mut-Reg1cp* or *WT-Reg1cp* plasmids (Fig. 3D). To further evaluate the effects of *Mut-Reg1cp* on β cells, we assessed the RNA level of MIN6 cells, which were transfected with *Mut-Reg1cp* or *WT-Reg1cp* plasmids. We then performed KEGG-enrichment analysis of the RNA-seq data and found that multiple β cell function related systems, including insulin secretion and cAMP signaling pathway were changed in *Mut-Reg1cp* plasmid transfected MIN6 cells compared with blank control or *WT-Reg1cp* plasmid transfected groups (Fig. 3E, F, Supplementary Fig. 3A, B). These results indicated that *Mut-Reg1cp* impaired the function of β cells.

### *Mut-Reg1cp* impaired the insulin secretion by inhibiting phosphorylation of PTBP1

*Reg1cp* belongs to *Reg* family and has been reported to promote colorectal cancer cell proliferation through activation of REG3A in colon cells [21]. However, we didn’t detect the changes in expression levels of *Reg1, Reg2, Reg3α* and *Reg3γ* in MIN6 cells transfected with *Mut-Reg1cp* or *WT-Reg1cp* plasmids (Supplementary Fig. 4).

Our previous study demonstrated that this mutation in *Reg1cp* led to a large change in its structure so that mutated *Reg1cp* could directly bind to KLF3 in endothelial cells to abolish the function of KLF3 [17]. To further elucidate the mechanisms whereby mutated *Reg1cp* may accelerate the development of T2D, we conducted mass spectrometry (MS) of proteins after RNA pulldown using *WT-Reg1cp* or *Mut-Reg1cp* in MIN6 cells. 16 polypeptides of 12 proteins could only be combined by *Mut-Reg1cp* instead of *WT-Reg1cp* (Supplementary Table 1). Among these identified peptides, 3 peptides from PTBP1 protein which could only be retrieved by *Mut-Reg1cp* caught our attentions (Supplementary Fig. 5A–D). cAMP-dependent phosphorylation of PTBP1 has been reported to be a common downstream target of glucose in the posttranscriptional upregulation of β cell’s secretory granule (SG) biogenesis [22]. So, we chose PTBP1 for further analysis.

**Fig. 4:**
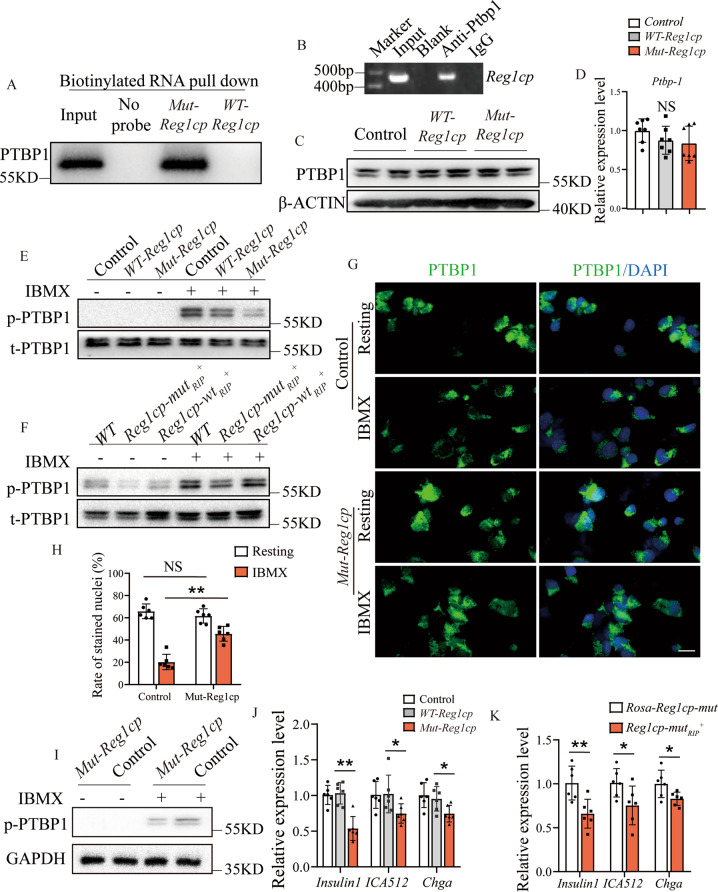
*Mut-Reg1cp* impaired the insulin secretion by inhibiting phosphorylation of PTBP-1. **A** *Mut-Reg1cp* retrieved PTBP1, as detected by immunoblotting. **B** Semi-quantitative PCR showed PTBP1 retrieved *Mut-Reg1cp*. **C**, **D** Western blot analysis of the expression of PTBP1 (**C**) and qRT-PCR analysis of *Ptbp1* (**D**) in MIN6 cells transfected with Control, *WT- Reg1cp* or *Mut-Reg1cp* plasmids. **E**, **F** Western blot analysis of the expression of phosphorylation and total PTBP1 (p-PTBP1 and t-PTBP1) in MIN6 cells transfected with Control, *WT- Reg1cp* or *Mut-Reg1cp* plasmids (**E**) or in islets isolated from *WT, Reg1cp-wt*_*RIP*_^*+*^or *Reg1cp-mut*_*RIP*_^*+*^ mice (**F**) with or without IBMX treatment. **G**, **H** Representative immunostaining images of PTBP1 (green) and DAPI, nucleus (blue) (**G**) and quantification of PTBP1 positive nucleus (**H**) in MIN6 cells transfected with Control o*r Mut-Reg1cp* plasmids before or after IBMX treatment. Scale bar: 20 μm. **I** Western blot analysis of the expression of p-PTBP1 in cytoplasm of MIN6 cells transfected with Control or *Mut-Reg1cp* plasmids with or without IBMX treatment. **J** RT-PCR analysis of *Insulin, ICA512* and *Chga* in MIN6 cells transfected with Control, *WT- Reg1cp* or *Mut-Reg1cp* plasmids. **K** RT-PCR analysis of *Insulin, ICA512* and *Chga* in islets isolated from *Rosa-Reg1cp-mut* or *Reg1cp-mut*_*RIP*_^*+*^ mice. **A**, **B** was representative of two independent experiments. In **C**–**K**, *n* = 6 in each group from two independent experiments. Data shown as mean ± SD. *P < 0.05; **P < 0.01; NS no significance; One-way ANOVA.

Biotinylated RNA pull-down confirmed that *Mut-Reg1cp*, but not *WT-Reg1cp*, specifically retrieved PTBP1 (Fig. 4A). We then conducted RNA immunoprecipitation of MIN6 transfected with the *Mut-Reg1cp* plasmids. The anti-PTBP1 antibody also could pull down *Mut-Reg1cp* (Fig. 4B). MIN6 cells transfected with *WT-Reg1cp* or *Mut-Reg1cp* plasmid had similar protein and mRNA level of PTBP1 compared with control groups (Fig. 4C, D). However, the phosphorylation PTBP1 level of MIN6 cells transfected with *Mut-Reg1cp* after IBMX treatment was significant lower when compared with the controls (Fig. 4E), and there was no difference between *Mut-Reg1cp* plasmid transfection group and co-expression of *Mut-Reg1cp* and *WT-Reg1cp* group (Supplementary Fig. 5E and F). The decreased phosphorylation level of PTBP1 was further confirmed in islet isolated from *Reg1cp-mut*_*RIP*_^*+*^ mice (Fig. 4F). These results indicated that *Mut-Reg1cp* could directly bind to PTBP1 and inhibit its phosphorylation.

cAMP-dependent phosphorylation induced the nucleocytoplasmic translocation of PTBP1 to stabilize mRNAs encoding various SG proteins in β cells [22]. IBMX treatment significantly reduced the number of PTBP1 positive nuclei in MIN6 cells. However, this phenomenon was attenuated after *Mut-Reg1cp* plasmid transfection (Fig. 4G, H). The lower phosphorylation of PTBP1 was further confirmed in cytoplasm of *Mut-Reg1cp* plasmid transfected MIN6 cells (Fig. 4I). The levels of mRNAs encoding components of SG proteins including *Insulin 1, pro-ICA512* and *Chga* in *Mut-Reg1cp* transfected MIN6 cells were lower after IBMX-stimulation (Fig. 4J). The lower level of *insulin 1, pro-ICA512* and *Chga* mRNAs were further confirmed in islet isolated from *Reg1cp-mut*_*RIP*_^*+*^ mice (Fig. 4K).

Taken together, these results indicated that *Mut-Reg1cp* attenuated the stabilization and translation of mRNAs encoding insulin via directly binding to and inhibiting the phosphorylation of PTBP1, which further impaired β cell function.

### Major metabolic tissues of *Reg1cp-mut*_*RIP*_^*+*^ mice obtained islets-derived exosomal *Reg1cp* and showed insulin resistance

**Fig. 5:**
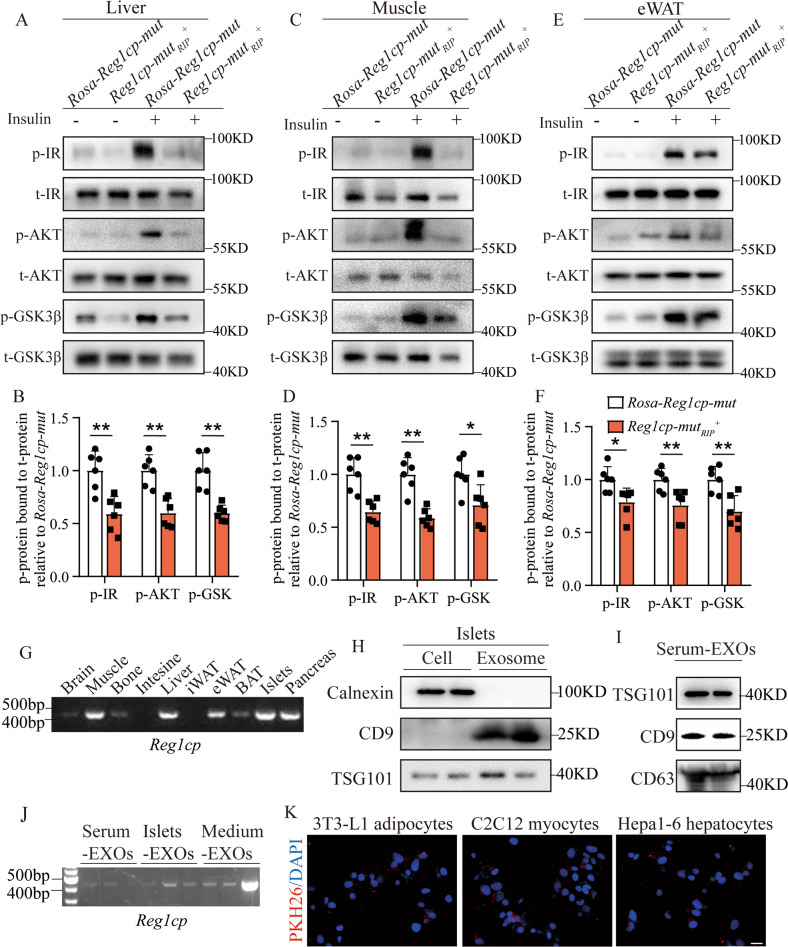
Major metabolic tissues of *Reg1cp-mut*_*RIP*_^*+*^ mice obtained islets-derived exosomal *Reg1cp* and showed insulin resistance. **A**–**F** Representative pictures (**A**, **C**, **E**) and quantitative measurements (**B**, **D**, **F**) of western blot analysis of insulin stimulated IR, AKT, and GSK3β phosphorylation in liver (**A**, **B**), muscle (**C**, **D**) and epididymal white adipose tissue (eWAT, **E**, **F**) of *Reg1cp-mut*_*RIP*_^*+*^ mice and related *Rosa-Reg1cp-mut* controls. **G** Semi-quantitative PCR showed the expression level of *Mut-Reg1cp* in different tissues of *Reg1cp-mut*_*RIP*_^*+*^ mice. **H** Protein levels of Calnexin, CD9 and TSG101 in MIN6 cells or exosomes released by MIN6 cells. **I** Protein levels of TSG101, CD9 and CD63 in exosomes isolated from mice serum. **J** Semi-quantitative PCR showed the expression level of *Mut-Reg1cp* in exosomes isolated from *Reg1cp-mut*_*RIP*_^*+*^ mice serum, cultured medium of primary islets isolated from *Reg1cp-mut*_*RIP*_^*+*^ mice or cultured medium of MIN6 cells transfected with *Mut-Reg1cp* plasmids. **K** MIN6 cells derived exosomes were marked with red fluorescence dye PKH26 and then cocultured with 3T3-L1 adipocytes, C2C12 myocytes, and Hepa1-6 hepatocytes; Representative immunostaining images of PKH26 (red) and DAPI, nucleus (blue). Scale bar: 20 μm. In **A**–**F**, *n* = 6 in each group from three independent experiments. **G**–**K** were representative of two independent experiments. Data shown as mean ± SD. *P < 0.05; **P < 0.01; Student’s *t* test.

*Reg1cp-mut*_*RIP*_^*+*^ mice showed significant increasing insulin resistance indicated by ITTs (Fig. 2H, I, L, M). Furthermore, the activation of insulin signaling in liver, muscle and epididymal white adipose tissue (ewat) of *Reg1cp-mut*_*RIP*_^*+*^ mice induced by insulin stimulations was also restrained when compared with the littermate controls as evidenced by the immunostaining of phospho (p)-IR, p-AKT, and p-GSK3β in these tissues (Fig. 5A–F). These results suggested that *Reg1cp-mut*_*RIP*_^*+*^ mice had significant impaired insulin sensitivity which couldn’t be explained by modulating phosphorylation of PTBP1 in β cells.

To further investigate the potential reasons for the significant increased insulin resistance in *Reg1cp-mut*_*RIP*_^*+*^ mice, we analyzed circulating proteins in serum from *Reg1cp-wt*_*RIP*_^*+*^ mice, *Reg1cp-mut*_*RIP*_^*+*^ mice and *wild type* mice after 12-week HFD through mass spectrometry. Thirteen identified proteins showed significant changes in *Reg1cp-mut*_*RIP*_^*+*^ mice compared with *Reg1cp-wt*_*RIP*_^*+*^ group and *wild type* control group (Supplementary Fig. 6A–E). None of those circulating proteins had been reported as an inducer of insulin resistance, except for CHGA, a secretory protein that occurs in endocrine, neuroendocrine, and neuronal cells and is also a major cargo in insulin secretory vesicles in β cells [23–25], whose expression level was affected by the cytoplasmic localization of PTBP1 [22]. We also confirmed the lower level of *Chga* mRNA in β cells isolated from *Reg1cp-mut*_*RIP*_^*+*^ mice or MIN6 cells transfected with *Mut-Reg1cp* plasmids (Fig. 4J, K). Thus, the higher circulation level of CHGA in *Reg1cp-mut*_*RIP*_^*+*^ mice (Supplementary Fig. 6C–E) might be cause by the hormone imbalance induced by insulin resistance. Those results indicated that the significant increased insulin resistance of metabolic tissues of *Reg1cp-mut*_*RIP*_^*+*^ mice might not due to the variation of circulating proteins in *Reg1cp-mut*_*RIP*_^*+*^ mice.

The *RIP-Cre* system which was supposedly specifically expressed Cre recombinase in 80% or more of β cells, has been reported with leaky expression in the brain and other neuroendocrine cell types such as the pituitary gland, not in other metabolic tissues [20]. It’s worth noting that, although the amount was very small, we detected the presence of *Reg1cp* in peripheral tissue such as muscle, liver and adipose tissue in transgenic mice (Fig. 5G, Supplementary Fig. 1A, B). Given that pancreatic islet could secret endocrine factors via exosomes to communicate with peripheral organs [26, 27], we investigated whether pancreatic islet-secreted exosomes would transfer *Mut-Reg1cp* into peripheral tissue. We isolated exosomes from cultured primary islets and serum of *Reg1cp-mut*_*RIP*_^*+*^ mice (Fig. 5H, I) and detected the presence of *Mut-Reg1cp* in those isolated exosomes (Fig. 5J). We also detected the presence of *Mut-Reg1cp* in exosomes isolated from culture medium of MIN6 cells transfected with *Mut-Reg1cp* plasmids (Fig. 5J). MIN6 cells derived exosomes were marked with red fluorescent dye PKH26 and then cocultured with 3T3-L1 adipocytes, C2C12 myocytes, or Hepa1-6 hepatocytes. Twelve hours later, these cells exhibited efficient uptake of MIN6 cells exosomes, as evidenced by the existence of red fluorescence inside these cells (Fig. 5K). All of those results indicated that islet in *Reg1cp-mut*_*RIP*_^*+*^ mice transferred the mutated *Reg1cp* into peripheral tissues via exosomes.

### Exosomal *Mut-Reg1cp* impaired insulin sensitivity in vivo

**Fig. 6:**
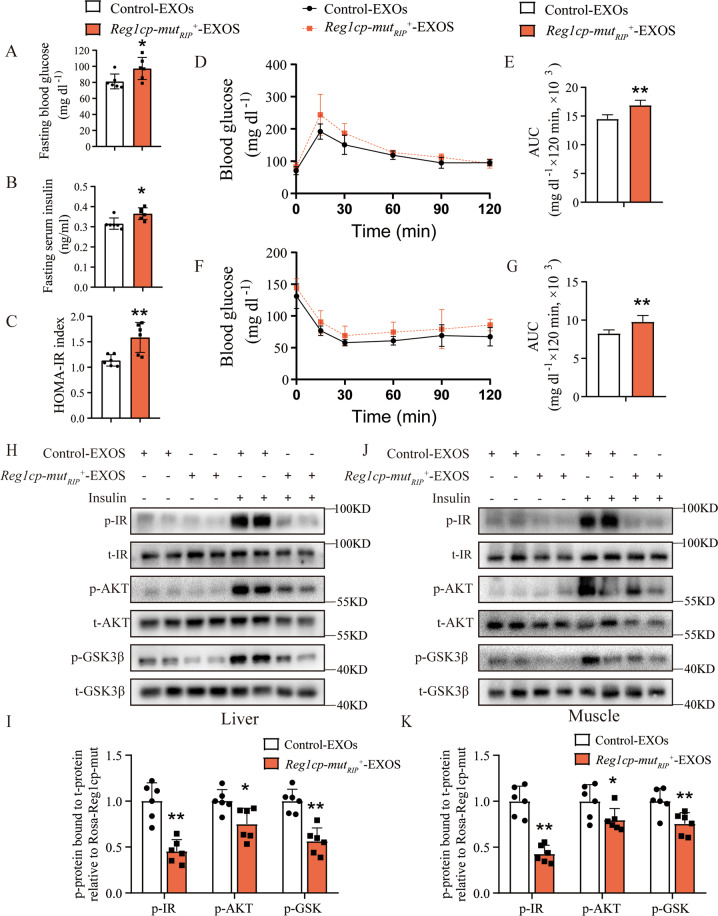
Exosomal *Mut-Reg1cp* impaired insulin sensitivity in vivo. **A**–**C** Fasting blood glucose level (**A**), fasting serum insulin level (**B**) and HOMA-IR index (**C**) of mice treated with exosomes isolated from serum of *Reg1cp-mut*_*RIP*_^*+*^ mice and *Rosa-Reg1cp-mut* controls. **D**–**G** The GTTs (**D**) and ITTs (**F**) in mice treated with serum exosomes isolated from *Reg1cp-mut*_*RIP*_^*+*^ mice and *Rosa-Reg1cp-mut* controls. Bar graphs showing the calculated values for area under curve (AUC) during the 120-min test course of the GTTs (**E**) and ITTs (**G**) (mg dl^−1^ × 120 min × 10^3^). **H**–**I** Representative pictures (**H**) and quantitative measurements (**I**) of Western blot analysis of insulin stimulated IR, AKT, and GSK3β phosphorylation in liver of mice treated with serum exosomes isolated from *Reg1cp-mut*_*RIP*_^*+*^ mice and *Rosa-Reg1cp-mut* controls. **J**, **K** Representative pictures (**J**) and quantitative measurements (**K**) of Western blot analysis of insulin stimulated IR, AKT, and GSK3β phosphorylation in muscle of mice treated with serum exosomes isolated from *Reg1cp-mut*_*RIP*_^*+*^ mice and *Rosa-Reg1cp-mut* controls. *n* = 6 in each group from three independent experiments. Data shown as mean ± SD. *P < 0.05; **P < 0.01; Student’s *t* test.

To further confirm the influences of islets-derived exosomes on insulin sensitivity in vivo, insulin-sensitive young *wild-type C57/BL6J* mice (2-month old) were administered with the exosomes isolated from serum of *Reg1cp-mut*_*RIP*_^*+*^ mice or *Rosa-Reg1cp-mut* controls via tail vein injection (two times per week for two weeks, 50 μg each time). Administration of circulating exosomes from *Reg1cp-mut*_*RIP*_^*+*^ mice increased the fasting blood glucose levels, fasting serum insulin, and HOMA-IR index of young *wild-type C57/BL6J* mice when compared with administration of circulating exosomes from *Rosa-Reg1cp-mut* control mice (Fig. 6A−C). Consistently, the glucose tolerance and clearance of young *wild-type C57/BL6J* mice were also significantly abrogated by the treatment of *Reg1cp-mut*_*RIP*_^*+*^ mice circulating exosomes, as shown by the results of GTTs and ITTs (Fig. 6D–G). Furthermore, the activation of insulin signaling in muscle and liver of young *wild-type C57/BL6J* mice induced by insulin stimulations was also inhibited by the treatment of *Reg1cp-mut*_*RIP*_^*+*^ mice circulating exosomes, as evidenced by the immunostaining of p-IR, p-AKT, and p-GSK3β in these tissues (Fig. 6H–K). Taken together, these results suggested that exosomes contained *Mut-Reg1cp* impaired insulin sensitivity in vivo.

### *Mut-Reg1cp* promoted insulin resistance by inhibiting AdipoR1 translation

Our previous study showed that *Mut-Reg1cp*, but not *WT-Reg1cp*, specifically bound to PTBP1 (Fig. 4A), which has been reported to play an important role in regulating ADIPOR1 protein expression and adiponectin signaling by associating with 3′UTR of AdipoR1 mRNA [28]. Downregulation of the AdipoR1 was involved in the development of insulin resistance and diabetes [29].

**Fig. 7:**
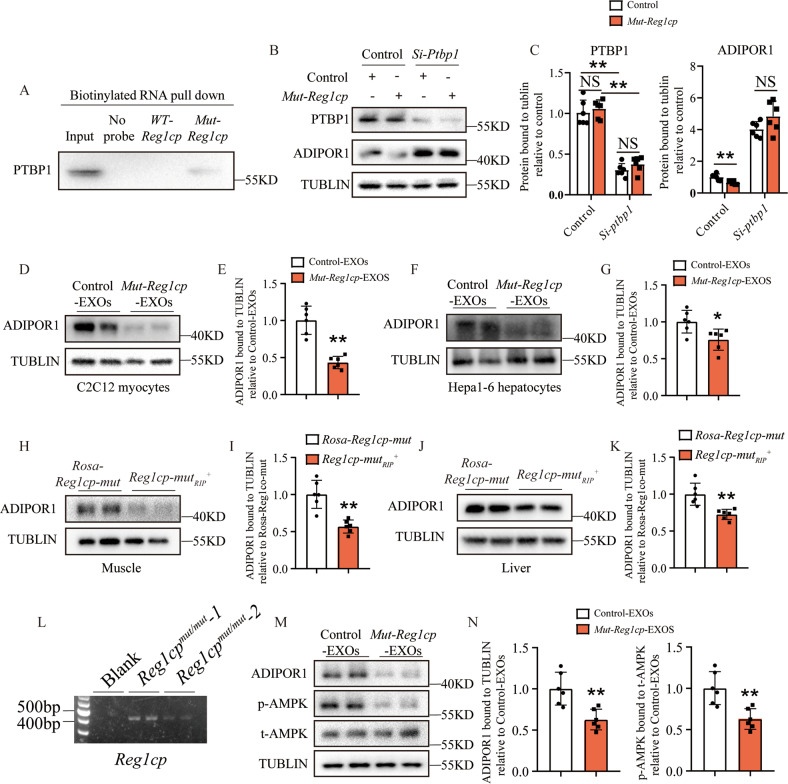
*Mut-Reg1cp* promoted insulin resistance by inhibiting AdipoR1 translation. **A** *Mut-Reg1cp* retrieved PTBP1, as detected by immunoblotting. **B**, **C** Representative pictures (**B**) and quantitative measurements (**C**) of western blot analysis of PTBP1 and ADIPOR1 level in C2C12 myocytes transfected with *Mut-Reg1cp* or control plasmids with or without si-Ptbp1 treatment. **D**, **E** Representative pictures (**D**) and quantitative measurements (**E**) of western blot analysis of ADIPOR1 level in C2C12 myocytes treated with exosomes contained *Mut-Reg1cp* or not. **F**, **G** Representative pictures (**F**) and quantitative measurements (**G**) of western blot analysis of ADIPOR1 level in Hepa1-6 hepatocytes treated with exosomes contained *Mut-Reg1cp* or not. **H**, **I** Representative pictures (**H**) and quantitative measurements (**I**) of western blot analysis of ADIPOR1 level in muscle of *Reg1cp-mut*_*RIP*_^*+*^ mice and *Rosa-Reg1cp-mut* controls. **J**, **K** Representative pictures (**J**) and quantitative measurements (**K**) of western blot analysis of ADIPOR1 level in liver of *Reg1cp-mut*_*RIP*_^*+*^ mice and *Rosa-Reg1cp-mut* controls. **L** Semi-quantitative PCR showed the expression level of *Reg1cp* in exosomes isolated from human serum. **M**, **N** Representative pictures (**M**) and quantitative measurements (**N**) of western blot analysis of ADIPOR1, p-AMPK and t-AMPK level in HepG2 hepatocytes treated with exosomes isolated from human serum. **A** and **L** were representative of two independent experiments. In **B**–**K**, **M**, **N**, *n* = 6 in each group from two independent experiments. Data shown as mean ± SD. *P < 0.05; **P < 0.01; NS no significance; Student’s *t* test for **E**, **G**, **I**, **K**, **N**. One-way ANOVA for **C**.

To further investigate whether *Mut-Reg1cp* impaired insulin sensitivity through PTBP1-AdipoR1 pathway. We transfected C2C12 myocytes with *Mut-Reg1cp* plasmid and confirmed the binding of *Mut-Reg1cp* and PTBP1 in C2C12 myocytes (Fig. 7A). We observed the lower expression level of ADIPOR1 (Fig. 7B, C) in C2C12 myocytes transfected with *Mut-Reg1cp* plasmid. However, the influence of *Mut-Reg1cp* on the expression of ADIPOR1 was disappeared after Ptbp1 siRNA treatment (Fig. 7B, C), which indicated that the *Mut-Reg1cp* impaired insulin sensitivity through PTBP1 dependent suppression on ADIPOR1 expression. The lower expression level of ADIPOR1 was also observed in C2C12 myocytes and Hepa1-6 hepatocytes treated with exosomes contained *Mut-Reg1cp* (Fig. 7D-G) or muscle and liver of *Reg1cp-mut*_*RIP*_^*+*^ mice (Fig. 7H–K). We also detected the presence of *Mut-Reg1cp* in exosomes isolated from serum of *Reg1cp*^*mut/mut*^ individuals (Fig. 7L). Furthermore, the treatment of exosomes isolated from serum of *Reg1cp*^*mut/mut*^ individuals decreased the level of ADIPOR1 and its downstream AMPK phosphorylation in human HepG2 hepatocytes (Fig. 7M, N). All of these results suggested that exosomes contained *Mut-Reg1cp* impaired insulin sensitivity through adiponectin signaling by inhibiting the expression of ADIPOR1 both in animal models and in human cells.

## Discussion

LncRNAs have been shown to be involved in controlling β-cell proliferation, apoptosis or function during T2D development [15, 16]. In the current study, we identified a mutation in *Reg1cp* and presented genetic, clinical, in vivo and in vitro experimental evidences to demonstrate that *Reg1cp* promotes the development of T2D both by impairing islet β cell function and increasing insulin resistance.

Regenerating genes (*Reg*) are the members of the calcium-dependent lectin gene superfamily [19, 30], which are involved in cell proliferation and differentiation [31, 32]. LncRNA *Reg1cp* is encoded as a pseudogene that is mainly expressed in the pancreas, especially in islet [18]. Our previous study identified this rs3819316 C > T mutation in *Reg1cp* through a HBM pedigree investigation [17]. As *Reg1cp* is mainly expressed in islet, we further investigated the function of *WT*-and *Mut-Reg1cp* in glucose homeostasis and found that *Mut-Reg1cp* individuals had higher incidence of T2D compared with *WT-Reg1cp* subjects and mice with islet-specific *Mut-Reg1cp* knock-in aggravated the disruption of glucose homeostasis, whereas *WT-Reg1cp* seemed to have no influence on glucose homeostasis. Hamed Yari et al. reported that *Reg1cp* promoted colorectal cancer cell proliferation through activation of REG3A in colon cells [21]. However, in this study we didn’t detect the changes in expression levels of *Reg1, Reg2, Reg3α* and *Reg3γ* in MIN6 cells transfected with *Mut-Reg1cp* or *WT-Reg1cp* plasmids, which indicated that *Reg1cp* performed different functions in different cells or tissues.

T2D develops when insulin secretion is unable to compensate for the increased insulin demand [33]. Pancreatic beta cell failure is the central event leading to the progression of insulin resistance to diabetes [33, 34]. In this study, we demonstrated that the abnormal glucose metabolism in islet-specific *Mut-Reg1cp* knock-in mice was attributed to impaired β cell function, rather than decreased β cell mass. LncRNAs regulate a wide range of biological processes in β cells in diverse mechanisms at the transcriptional level, post-transcriptional level, and post-translational level by binding with DNA, RNA, or protein complexes [15]. This identified mutation in *Reg1cp* did not affect its expression but led to a large change in its structure [17]. Previously we demonstrated that *Reg1cp* was involved in HBM pathogenesis by directly binding to KLF3 to abolish its function in endothelial cells [17]. However, we didn’t detect the binding of KLF3 and *WT-* or *Mut-Reg1cp* in MIN6 cells in this study, but we detected 3 peptides from PTBP1 protein could be retrieved by *Mut-Reg1cp* rather than *WT-Reg1cp* and the mutant *Reg1cp* did not compete with the *WT* form.

PTBP1, a ubiquitous RNA-binding protein, which belongs to polypyrimidine-tract-binding protein family, functions in diverse cellular processes including mRNA stability and translation initiation [35, 36]. Specificity of PTBP1 function is achieved by its cellular localization. Klaus-Peter Knoch et al. have reported that PTBP1 is a common downstream target of glucose and GLP-1 in upregulating β cell’s proinsulin biogenesis by nucleocytoplasmic translocation [22, 37], and the nucleocytosolic translocation of PTBP1 is relay on the phosphorylation on serine 16 [22, 37]. Florian Ehehalt et al. observed the decreased nuclear level of PTBP1 in stimulated non-diabetic islets, but not in type 2 diabetic islets. They demonstrated that impaired redistribution of PTBP1 and deficient upregulation of insulin levels are characteristic traits of islets isolated from T2D patients [38]. In our study, we demonstrated that *Mut-Reg1cp* could bind to and inhibit the phosphorylation of PTBP1 and we also observed the restrained nucleocytosolic translocation of PTBP1 and lower insulin/pro-insulin mRNA level after cAMP stimulation in MIN6 cells, which indicated that the impaired β cell function in *Reg1cp-mut*_*RIP*_^*+*^ mice was due to blocked PTBP1 phosphorylation. We did not investigate whether *Mut-Reg1cp* is involved in other signaling pathways. However, this should be addressed in a future study.

It is widely accepted that islet β cell dysfunction and insulin resistance are central components in the etiology of T2D. We found that *Mut-Reg1cp* in islet β cells not only impaired β cell function, but also aggravated insulin resistance in mice. Exosomes are small extracellular vesicles with 30–120 nM in size, carrying diverse bioactive molecules that can act as endocrine factors and mediate the crosstalk between different tissues [39]. In recent years, researchers have found that pancreatic islet could secret endocrine factors via exosomes to communicate with peripheral organs. Islet-derived exosomes from diabetes patients contain dysregulated RNAs under cytokine stress [26]. Pancreatic islets could secrete exosomes that act in an autocrine manner to regulate cell proliferation and death [26, 27, 40]. Pancreatic β cells also have been demonstrated to control glucose homeostasis via the secretion of exosomal *miR-29* family and *miR-26a* [41, 42]. In our project, we demonstrated that islet-derived exosomes transferred the mutated *Reg1cp* into peripheral tissues such as liver and muscle and impaired insulin sensitivity through adiponectin signaling by inhibiting the expression of ADIPOR1. Our research indicated that peripheral blood exosomal-*Mut-Reg1cp* was an insulin resistance risk factor and could be used in early clinical diagnosis of T2D.

In summary, our work reveals a new mechanism of β cell dysfunction and insulin resistance in the development of T2D, suggesting that rs3819316 C > T mutation in *Reg1cp* can be added to the growing list of genes implicated in the risk of developing T2D and screening the *Reg1cp*^*+/mut*^ or *Reg1cp*^*mut/mut*^ individuals might be beneficial to early clinical diagnosis of T2D.

## Methods

### Human participants for genotyping and glucose metabolism related indexes analysis

Human participants were enrolled from Shanghai Nicheng community, China and Endocrinology Department of Xiangya Hospital of Central South University, Changsha, China. Individuals with missing glucose metabolism related indexes, severe disability, hepatic dysfunction and cancer were excluded. Subjects without a self-reported history of diabetes were administrated to an oral glucose tolerance test (OGTT) after a 75 g oral glucose load. Diabetes was diagnosed according to the 2010 criteria of the American Diabetes Association (ADA) [43].

Medical history, details of the anthropometric indices and biochemical traits related to diabetes were recorded. Height (m), weight (kg), waist circumference (cm) and hip circumference (cm) were measured, and body mass index (BMI) was calculated as weight/height^2^. Blood samples were collected for the measurements of metabolic indexes including glycated hemoglobin A1c (HbA1c). Plasma glucose and insulin levels at 0, 30, 120 min timepoints of all participants were also tested. Based on these collected indexes, area under the curve of the glucose from 0 to 120 min (GAUC) and area under the curve of the insulin from 0 to 120 min (IAUC) were calculated. Insulin resistance and β cell function were assessed by HOMA for insulin resistance index (HOMA-IR) and beta cell function (HOMA-β) [44]. Stumvoll first- and second-phase insulin secretion indices (STU1, STU2) and GUTT index were generated by computations proposed by Stumvoll et al. [45] and Gutt et al. [46].

The human study was approved by the Institutional Review Board of Shanghai Jiao Tong University Affiliated Sixth People’s Hospital, Shanghai, China and Ethics Committee of Xiangya Hospital of Central South University, Changsha, China. It was conducted in accordance with the principles of the Second Revision of the Declaration of Helsinki, and written informed consent was signed by every participant.

### Mice

To generate islet β cell specific *WT-Reg1cp* or *Mut-Reg1cp* knock-in mice, mice carrying a loxP-flanked-Stop cassette controlling *WT-Reg1cp* or *Mut-Reg1cp* alleles expression (*Rosa-Reg1cp-wt* mice or *Rosa-Reg1cp-mut* mice) and *RIP-Cre* transgenics were interbred. The *Rosa-Reg1cp-w*t mice or *Rosa-Reg1cp-mut* littermates were used as controls. The *RIP-Cre* transgenic mice (Stock No. C001002) were purchased from Cyagen Biosciences Inc (China). *Rosa-Reg1cp-wt* mice and *Rosa-Reg1cp-mut* mice were constructed by Bioray Laboratories Inc (China). For mice experiments, six male mice were used for each group for each independent experiment. Animal care protocols and experiments were reviewed and approved by Medical Ethics Committee of Xiangya Hospital of Central South University. All mice were maintained in the specific pathogen-free facility of the Department of Laboratory Animals, Central South University.

### Primary cell isolation and cell culture

Pancreatic islets were isolated from whole pancreas of mice through the injection of 0.8 mg/mL collagenase V (Sigma–Aldrich) into the pancreatic duct, followed by digestion at 37 °C for 25 min with mild shaking, and isolated islets were picked by hand selection under a dissecting microscope. Pancreatic islets were treated with 0.05% trypsin in PBS for 5 min at 37 °C to isolate primary pancreatic islet β-cells.

Pancreatic islets and MIN6 cells were cultured in RPMI 1640 medium containing 10% FBS, 1mM L-glutamine, 1 mM HEPES, 1 mM sodium pyruvate, 50 μM 2-mercaptoethanol, and 1% penicillin–streptomycin. 3T3-L1, C2C12, or HepG2, Hepa1-6 cells were cultured in DMEM (Gibco) containing 10% FBS and 1% penicillin–streptomycin. All cells were cultured in a humidified incubator with 95% air and 5% CO_2_ at 37 °C. MIN6 cell line was bought from MeisenCTCC company and has been authenticated by STR profiling.

### Exosomes isolation, identification and treatment

Serum exosomes were isolated using ExoQuick^TM^ Exosome Precipitation Solution (SBI, EXOQ5A-1) according to the manufactor’s instructions. Culture medium exosomes were isolated using Total Exosome Isolation (from cell culture media) Kit (Invitrogen, 4478359) according to the manufactor’s instructions. To identify the isolated exosomes, Western blotting analysis was conducted to detect the expression of TSG101 (Proteintech, 28283-1-AP, 1:1000), Calnexin (Proteintech, 10427-2-AP, 1:1000), CD63 (Santa Cruz Biotechnology, sc-5275, 1:1000), and CD9 (Proteintech, 20597-1-AP, 1:1000).

For the administration of exosomes to mice, exosomes were injected to recipient mice via tail vein injection (two times a week, 50 μg/time for 2 weeks). For the cell treatment, exosomes were added to the culture medium on the basis of 2 μg of exosomes per 1 × 10^5^ recipient cells. To monitor the exosome trafficking, exosomes were marked by a PKH26 fluorescent cell linker kit (Sigma), according to the manufactor’s instructions.

### Blood glucose, serum insulin and HOMA-IR index

The measurement of blood glucose levels and serum insulin were performed as reported previously [39]. Blood glucose concentrations were measured by glucometer. Serum Insulin levels were tested by Mouse Insulin (INS) Elisa Kit (Nanjing Jiancheng Bioengineering Institute, H203-1-2) according to manufacturer’s instructions.

### Glucose and insulin tolerance tests

The glucose tolerance tests (GTTs) and insulin tolerance tests (ITTs) were performed as reported previously [12]. For GTTs, mice were intraperitoneally treated with glucose at dosage of 1 g/kg after overnight fast. For ITTs, mice were intraperitoneally treated with insulin at dosage of 0.75 U/kg after 6 h fast. Blood was collected by venous bleeding from the tail vein at 0, 15, 30, 60, and 120 min after glucose or insulin injection, and glucose concentrations were measured by glucometer.

### GSIS

For MIN6 cell, cells were seeded onto 12-well plates at a density of 1 × 10^6^ cells. For primary islets, 30–40 islets were handpicked for each assay replicate per well. Medium was collected after a 1-h incubation at each glucose concentration. For in vivo GSIS, the insulin was measured from serum collected at the 0 and 15 min after an intraperitoneal injection of glucose (2 g/kg body weight). Secreted insulin levels were measured using an enzyme-linked immunosorbent assay (ELISA) insulin kit (Nanjing Jiancheng Bioengineering Institute, H203). Results were normalized to the total insulin content.

### Immunofluorescence staining

Immunofluorescence staining was performed as reported previously [47]. For tissue Immunohistochemistry staining, samples were embedded in paraffin. Four-micrometer-thick sections were stained with individual primary antibodies to mouse insulin (Servicebio, gb13121, 1:300), Glucagon (Servicebio, gb11097, 1:1000), Ki67 (Abcam, ab15580, 1:100), P21(Abcam, ab188224, 1:100) overnight at 4 °C. For Immunocytochemistry staining, cells were stained with individual primary antibodies to PTBP1 (Thermo Fisher/Invitrogen, 32-4800, 1:100) overnight at 4 °C. We counted the numbers of positively stained cells in four random visual fields in five sequential sections per sample in each group.

### SA-β-gal assay

MIN6 senescence was induced by treating with 450 μM H_2_O_2_ for 24 h and then cellular senescence was evaluated by senescence-associated beta-galactosidase (SA beta-gal) staining using a Senescence β-Galactosidase Staining Kit (Solarbio, G1580) according to manufacturer’s instructions.

### RNA sequencing and bioinformatics analysis

After RNA quality examination, a total amount of 2 μg RNA per sample was used as input material for the RNA sample preparations. Sequencing libraries were generated using NEBNext® Ultra™ RNA Library Prep Kit for Illumina® (#E7530L, NEB, USA) following the manufacturer’s recommendations and index codes were added to attribute sequences to each sample. Briefly, mRNA was purified from total RNA using poly-T oligo-attached magnetic beads. Fragmentation was carried out using divalent cations under elevated temperature in NEBNext First Strand Synthesis Reaction Buffer (5X). First strand cDNA was synthesized using random hexamer primer and RNase H. Second strand cDNA synthesis was subsequently performed using buffer, dNTPs, DNA polymerase I and RNase H. The library fragments were purified with QiaQuick PCR kits and elution with EB buffer, then terminal repair, A-tailing and adapter added were implemented. The aimed products were retrieved and PCR was performed, then the library was completed. The clustering of the index-coded samples was performed on a cBot cluster generation system using HiSeq PE Cluster Kit v4-cBot-HS (Illumina) according to the manufacturer’s instructions. After cluster generation, the libraries were sequenced on an Illumina platform and 150 bp paired-end reads were generated. KEGG Enrichment (fisher.test and p.adjust) was performed to show the biological processes affected among different groups. The RNA sequencing data was deposited on the Sequence Read Archive website with the BioProject accession number PRJNA874533.

### RNA pull-down assay

The 3’-end biotin-labeled RNA probes used in RNA pull-down were generated using RNA 30 End Biotinylation Kit (20160, Pierce) according to manufacturer’s instructions. Five picomoles of 30-biotinylated RNA was used in each pulldown assay. Briefly, biotin-labeled full-length *WT-Reg1cp* or *Mut-Reg1cp* were incubated with nuclear lysate of MIN6 or C2C12 cells for 60 min. Then, streptavidin agarose beads (Invitrogen) were added and incubated at room temperature (RT) for another 60 min. The retrieved proteins were subjected to western blot analysis or mass spectrometry. The mass spectrometry assay was performed by Shanghai Applied Protein Technology Co. Ltd.

### RNA immunoprecipitation

RNA immunoprecipitation was performed using Magna RIP RNA-Binding Protein Immunoprecipitation Kit (17-700, Millipore) according to manufacturer’s instructions. The RNA samples precipitate was extracted, reverse transcribed using T7 High YieldRNA Synthesis Kit (E2040S, NEB) according to manufacturer’s instructions and subjected to qRT-PCR analysis.

### Western blot

Protein samples were separated by SDS-PAGE (sodium dodecyl sulfate polyacrylamide gel electrophoresis) and blotted on polyvinylidene difluoride membranes (Millipore). The membranes were blocked with 5% milk (170-6404, Bio-Rad Laboratories, Inc.) and incubated with specific primary antibodies: PTBP1 (Thermo Fisher/Invitrogen, 32-4800, 1:1000), AdipoR1 (Santa Cruz Biotechnology, sc-518030,1:500), P-IR (Cell Signaling Technology, 3024S, 1:1000), t-IR (Cell Signaling Technology, 3025S, 1:1000), P-AKT (Cell Signaling Technology, 9271S, 1:1000), t-AKT (Cell Signaling Technology, 4691S, 1:1000), P-GSK3β (Cell Signaling Technology, 9336S, 1:1000), t-GSK3β (Cell Signaling Technology, 9315S, 1:1000), GAPDH (OriGene, TA802519, 1:3000), β-ACTIN (OriGene, TA811000, 1:3000), then reprobed with appropriate horseradish peroxidase–conjugated secondary antibodies. Blots were visualized using SuperSignal West Pico PLUS Chemiluminescent Substrate (SD251210, Thermo Fisher Scientific, Inc.) Phosphorylation PTBP1 antibody was constructed by ProMab Biotechnologies (Hunan China) used the antigen as a synthetic peptide GTKRGSDELF (PTB1 amino acids 11-20) with Ser-16 phosphorylated.

### Statistical analyses

Data are presented as mean ± standard deviation or median (interquartile range). For comparisons of two groups, two-tailed Student’s *t*-test was used. For comparisons of multiple groups, one-way analysis of variance (ANOVA) was used followed by Bonferroni’s posttest in GraphPad Prism 7.0. Differences were considered significant at *P* < 0.05. The clinical characteristics of the patients were summarized with SAS 9.2 (SAS Institute, Cary, NC, USA). For continuous variables, normality testing was performed, quantitative traits with skewed distribution were logarithmically transformed, mean ± standard deviation or median (interquartile range) was used for a general description, differences between groups were determined via the Wilcoxon test. For categorical variables, the number of subjects were used for a general description, and differences between groups were determined via the χ2 test. Associations between phenotypes and genotypes were determined in PLINK (v1.07;http://pngu.mgh.harvard. edu/~purcell/plink/) using an additive genetic model. The associations of rs3819316 C > T mutation with diabetes and prediabetic states were tested by a logistic regression model. Linear regression analysis was used to test the associations of rs3819316 C > T mutation with glucose metabolism related indexes. No randomization or blinding was used, and no animals were excluded from analysis. Sample sizes were selected on the basis of previous experiments.

## Supplementary information

supplementary figures

Supplementary Table 1

Full uncut gels

Reproducibility checklist

## Data Availability

The data that support the findings of this study are available within the article and Supplementary Files or available from the corresponding authors upon reasonable request. Microarray data that support the findings of this study have been deposited in Sequence Read Archive website (Home - SRA - NCBI (nih.gov)) with the BioProject accession number PRJNA874533.

## References

[1] International Diabetes Federation. IDF Diabetes Atlas, 10th edn. 2021. http://www.diabetesatlas.org/ (2021). 35914061

[2] Sun H, Saeedi P, Karuranga S, Pinkepank M, Ogurtsova K, Duncan BB (2022). IDF Diabetes Atlas: Global, regional and country-level diabetes prevalence estimates for 2021 and projections for 2045. Diabetes Res Clin Pr.

[3] Zheng Y, Ley SH, Hu FB (2018). Global aetiology and epidemiology of type 2 diabetes mellitus and its complications. Nat Rev Endocrinol.

[4] Holman N, Young B, Gadsby R (2015). Current prevalence of type 1 and type 2 diabetes in adults and children in the UK. Diabet Med.

[5] Khetan S, Kales S, Kursawe R, Jillette A, Ulirsch JC, Reilly SK (2021). Functional characterization of T2D-associated SNP effects on baseline and ER stress-responsive beta cell transcriptional activation. Nat Commun.

[6] de Goede OM, Nachun DC, Ferraro NM, Gloudemans MJ, Rao AS, Smail C (2021). Population-scale tissue transcriptomics maps long non-coding RNAs to complex disease. Cell.

[7] Cho YS, Chen CH, Hu C, Long J, Ong RT, Sim X (2011). Meta-analysis of genome-wide association studies identifies eight new loci for type 2 diabetes in east Asians. Nat Genet.

[8] Yan J, Jiang F, Zhang R, Xu T, Zhou Z, Ren W (2017). Whole-exome sequencing identifies a novel INS mutation causative of maturity-onset diabetes of the young 10. J Mol Cell Biol.

[9] Knoll M, Lodish HF, Sun L (2015). Long non-coding RNAs as regulators of the endocrine system. Nat Rev Endocrinol.

[10] Akerman I, Tu Z, Beucher A, Rolando DMY, Sauty-Colace C, Benazra M (2017). Human pancreatic beta cell lncRNAs control cell-specific regulatory networks. Cell Metab.

[11] Li CJ, Xiao Y, Yang M, Su T, Sun X, Guo Q (2018). Long noncoding RNA Bmncr regulates mesenchymal stem cell fate during skeletal aging. J Clin Invest.

[12] Xiao YZ, Yang M, Xiao Y, Guo Q, Huang Y, Li CJ (2020). Reducing hypothalamic stem cell senescence protects against aging-associated physiological decline. Cell Metab.

[13] Wong CM, Tsang FH, Ng IO (2018). Non-coding RNAs in hepatocellular carcinoma: molecular functions and pathological implications. Nat Rev Gastroenterol Hepatol.

[14] Ransohoff JD, Wei Y, Khavari PA (2018). The functions and unique features of long intergenic non-coding RNA. Nat Rev Mol Cell Biol.

[15] Lopez-Noriega L, Rutter GA (2020). Long non-coding RNAs as key modulators of pancreatic beta-cell mass and function. Front Endocrinol.

[16] Mirza AH, Kaur S, Pociot F (2017). Long non-coding RNAs as novel players in beta cell function and type 1 diabetes. Hum Genomics.

[17] Yang M, Guo Q, Peng H, Xiao YZ, Xiao Y, Huang Y (2019). Kruppel-like factor 3 inhibition by mutated lncRNA Reg1cp results in human high bone mass syndrome. J Exp Med.

[18] Gharib B, Fox MF, Bartoli C, Giorgi D, Sansonetti A, Swallow DM (1993). Human regeneration protein/lithostathine genes map to chromosome 2p12. Ann Hum Genet.

[19] Miyashita H, Nakagawara K, Mori M, Narushima Y, Noguchi N, Moriizumi S (1995). Human REG family genes are tandemly ordered in a 95-kilobase region of chromosome 2p12. FEBS Lett.

[20] Magnuson MA, Osipovich AB (2013). Pancreas-specific Cre driver lines and considerations for their prudent use. Cell Metab.

[21] Yari H, Jin L, Teng L, Wang Y, Wu Y, Liu GZ (2019). LncRNA REG1CP promotes tumorigenesis through an enhancer complex to recruit FANCJ helicase for REG3A transcription. Nat Commun.

[22] Knoch KP, Meisterfeld R, Kersting S, Bergert H, Altkruger A, Wegbrod C (2006). cAMP-dependent phosphorylation of PTB1 promotes the expression of insulin secretory granule proteins in beta cells. Cell Metab.

[23] Bandyopadhyay GK, Mahata SK. Chromogranin A regulation of obesity and peripheral insulin sensitivity. Front Endocrinol. 2017;8:20. 10.3389/fendo.2017.00020 PMC5296320 28228748

[24] Ying W, Riopel M, Bandyopadhyay G, Dong Y, Birmingham A, Seo JB (2017). Adipose tissue macrophage-derived exosomal miRNAs can modulate in vivo and in vitro insulin sensitivity. Cell..

[25] Taupenot L, Harper KL, O’Connor DT (2003). The chromogranin-secretogranin family. N. Engl J Med.

[26] Krishnan P, Syed F, Jiyun Kang N, Mirmira RG, Evans-Molina C (2019). Profiling of RNAs from human islet-derived exosomes in a model of type 1 diabetes. Int J Mol Sci.

[27] Xiong L, Chen L, Wu L, He W, Chen D, Peng Z (2022). Lipotoxicity-induced circGlis3 impairs beta cell function and is transmitted by exosomes to promote islet endothelial cell dysfunction. Diabetologia..

[28] Lustig Y, Barhod E, Ashwal-Fluss R, Gordin R, Shomron N, Baruch-Umansky K (2014). RNA-binding protein PTB and microRNA-221 coregulate AdipoR1 translation and adiponectin signaling. Diabetes..

[29] Yamauchi T, Nio Y, Maki T, Kobayashi M, Takazawa T, Iwabu M (2007). Targeted disruption of AdipoR1 and AdipoR2 causes abrogation of adiponectin binding and metabolic actions. Nat Med.

[30] Zhang YW, Ding LS, Lai MD (2003). Reg gene family and human diseases. World J Gastroenterol.

[31] Kiji T, Dohi Y, Nishizaki K, Takasawa S, Okamoto H, Nagasaka S (2003). Enhancement of cell viability in cryopreserved rat vascular grafts by administration of regenerating gene (REG) inducers. J Vasc Res.

[32] Tohma Y, Dohi Y, Shobatake R, Uchiyama T, Takeda M, Takasawa S, et al. Reg gene expression in periosteum after fracture and its in vitro induction triggered by IL-6. Int J Mol Sci. 2017;18:2257 10.3390/ijms18112257 PMC5713227 29077068

[33] So WY, Liu WN, Teo AKK, Rutter GA, Han W. Paired box 6 programs essential exocytotic genes in the regulation of glucose-stimulated insulin secretion and glucose homeostasis. Sci Transl Med. 2021;13:eabb1038. 10.1126/scitranslmed.abb1038 34193609

[34] Bell GI, Polonsky KS (2001). Diabetes mellitus and genetically programmed defects in beta-cell function. Nature..

[35] Sawicka K, Bushell M, Spriggs KA, Willis AE (2008). Polypyrimidine-tract-binding protein: a multifunctional RNA-binding protein. Biochem Soc Trans.

[36] Knoch KP, Nath-Sain S, Petzold A, Schneider H, Beck M, Wegbrod C (2014). PTBP1 is required for glucose-stimulated cap-independent translation of insulin granule proteins and Coxsackieviruses in beta cells. Mol Metab.

[37] Xie J, Lee JA, Kress TL, Mowry KL, Black DL (2003). Protein kinase A phosphorylation modulates transport of the polypyrimidine tract-binding protein. Proc Natl Acad Sci USA.

[38] Ehehalt F, Knoch K, Erdmann K, Krautz C, Jager M, Steffen A (2010). Impaired insulin turnover in islets from type 2 diabetic patients. Islets..

[39] Su T, Xiao Y, Xiao Y, Guo Q, Li C, Huang Y (2019). Bone marrow mesenchymal stem cells-derived exosomal MiR-29b-3p regulates aging-associated insulin resistance. ACS Nano.

[40] Ribeiro D, Horvath I, Heath N, Hicks R, Forslow A, Wittung-Stafshede P (2017). Extracellular vesicles from human pancreatic islets suppress human islet amyloid polypeptide amyloid formation. Proc Natl Acad Sci USA.

[41] Xu H, Du X, Xu J, Zhang Y, Tian Y, Liu G (2020). Pancreatic beta cell microRNA-26a alleviates type 2 diabetes by improving peripheral insulin sensitivity and preserving beta cell function. PLoS Biol.

[42] Li J, Zhang Y, Ye Y, Li D, Liu Y, Lee E (2021). Pancreatic beta cells control glucose homeostasis via the secretion of exosomal miR-29 family. J Extracell Vesicles.

[43] American Diabetes A. (2010). Diagnosis and classification of diabetes mellitus. Diabetes Care..

[44] Matthews DR, Hosker JP, Rudenski AS, Naylor BA, Treacher DF, Turner RC (1985). Homeostasis model assessment: insulin resistance and beta-cell function from fasting plasma glucose and insulin concentrations in man. Diabetologia..

[45] Stumvoll M, Van Haeften T, Fritsche A, Gerich J (2001). Oral glucose tolerance test indexes for insulin sensitivity and secretion based on various availabilities of sampling times. Diabetes Care.

[46] Gutt M, Davis CL, Spitzer SB, Llabre MM, Kumar M, Czarnecki EM (2000). Validation of the insulin sensitivity index (ISI0,120): comparison with other measures. Diabetes Res Clin Pract.

[47] Yang M, Li CJ, Sun X, Guo Q, Xiao Y, Su T (2017). MiR-497 approximately 195 cluster regulates angiogenesis during coupling with osteogenesis by maintaining endothelial Notch and HIF-1alpha activity. Nat Commun.

