# Correction: Mutated lncRNA increase the risk of type 2 diabetes by promoting β cell dysfunction and insulin resistance

**DOI:** 10.1038/s41419-022-05467-4

**Published:** 2022-11-30

**Authors:** Wan-Hui Guo, Qi Guo, Ya-Lin Liu, Dan-Dan Yan, Li Jin, Rong Zhang, Jing Yan, Xiang-Hang Luo, Mi Yang

**Affiliations:** 1 Department of Endocrinology, Endocrinology Research Center, Xiangya Hospital of Central South University, 410008 Changsha, Hunan P.R. China; 2 National Clinical Research Center for Geriatric Disorders, Xiangya Hospital, 410008 Changsha, Hunan P.R. China; 3 Shanghai Diabetes Institute, Shanghai Key Laboratory of Diabetes Mellitus, Shanghai Clinical Centre for Diabetes, Shanghai Sixth People’s Hospital affiliated to Shanghai Jiao Tong University School of Medicine, 200233 Shanghai, P.R. China

**Keywords:** Mechanisms of disease, Long non-coding RNAs, Type 2 diabetes, Type 2 diabetes, Type 2 diabetes

Correction to: *Cell Death and Disease* 10.1038/s41419-022-05348-w, published online 27 October 2022

The original version of this article contained a mistake. In this published article, an incorrect citation was mistakenly used in the 6th reference (Kovarovic K, Andrews P. Bovid postcranial ecomorphological survey of the Laetoli paleoenvironment. J Hum Evol. 2007; 52:663–80.). The error was made during the editing stage with some problems of the reference insert software, and this error was not detected during the subsequent proofreading stage. The correct citation of the 6th reference should be “de Goede OM, Nachun DC, Ferraro NM, Gloudemans MJ, Rao AS, Smail C, et al. Population-scale tissue transcriptomics maps long non-coding RNAs to complex disease. Cell. 2021;184:2633–48.e19.” The original article has been corrected.

