# Supplementary material for: Mutated lncRNA increase the risk of type 2 diabetes by promoting β cell dysfunction and insulin resistance
Source: Cell Death Dis. 2022 Oct 27;13(10):904. doi: 10.1038/s41419-022-05348-w (PMC9613878; doi:10.1038/s41419-022-05348-w)
Supplement: Supplementary file 1 — supplementary figures [file 41419_2022_5348_MOESM1_ESM.pdf]

Supplementary Figure 1

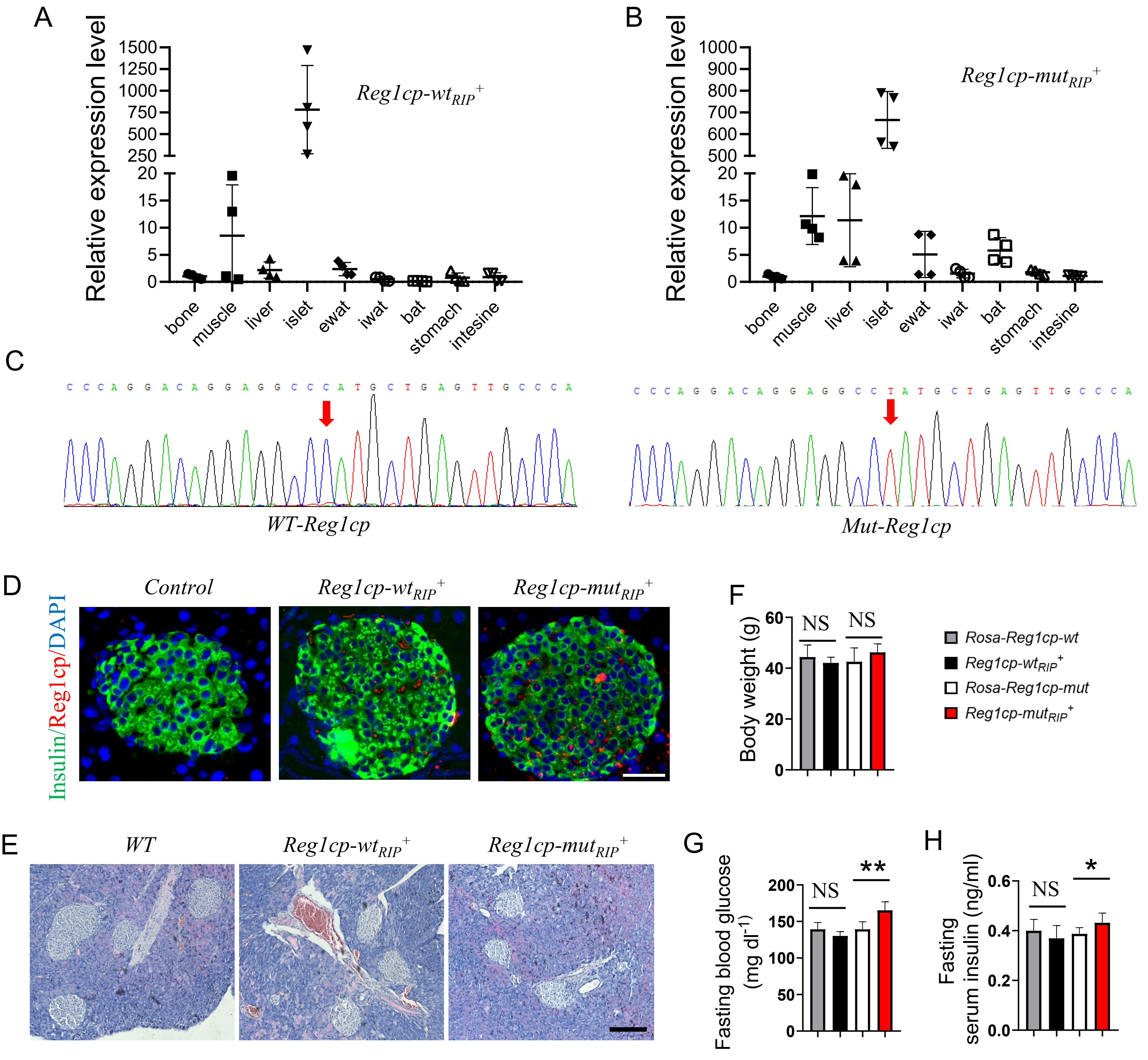

Supplementary Figure 1.

Insulin positive islet β cells specific *WT-Reg1cp* or *Mut-Reg1cp* knock-in mice models were successfully constructed

(A) RT-PCR analysis of the expression level of *WT-Reg1cp* in different tissues of *Reg1cp-wt*$_{RIP}^{+}$ mice. (B) RT-PCR analysis of the expression level of *Mut-Reg1cp* in different tissues of *Reg1cp-mut*$_{RIP}^{+}$ mice. (C) Sanger sequencing results of *Reg1cp* transcripts from *Reg1cp-wt*$_{RIP}^{+}$ or *Reg1cp-mut*$_{RIP}^{+}$ mice. (D) Representative image of insulin (green) and *Reg1cp* (red) staining of islets in *Reg1cp-wt*$_{RIP}^{+}$, *Reg1cp-mut*$_{RIP}^{+}$ or Control mice. Scale bar: 50μm. (E) Representative image of HE staining of pancreas of *Reg1cp-wt*$_{RIP}^{+}$, *Reg1cp-mut*$_{RIP}^{+}$ or *wild-type C57/BL6J* mice. Scale bar: 200μm. (F-H) The body weight (F), fasting blood glucose level (G) and fasting serum insulin level (H) of *Rosa-Reg1cp-wt*, *Rosa-Reg1cp-mut*, *Reg1cp-wt*$_{RIP}^{+}$ and *Reg1cp-mut*$_{RIP}^{+}$ mice under HFD. n = 4 in each group from three independent experiments. Data shown as mean ± SD. **, $P < 0.01$; *, $P < 0.05$; NS, no significance; One-way ANOVA.

Supplementary Figure 2

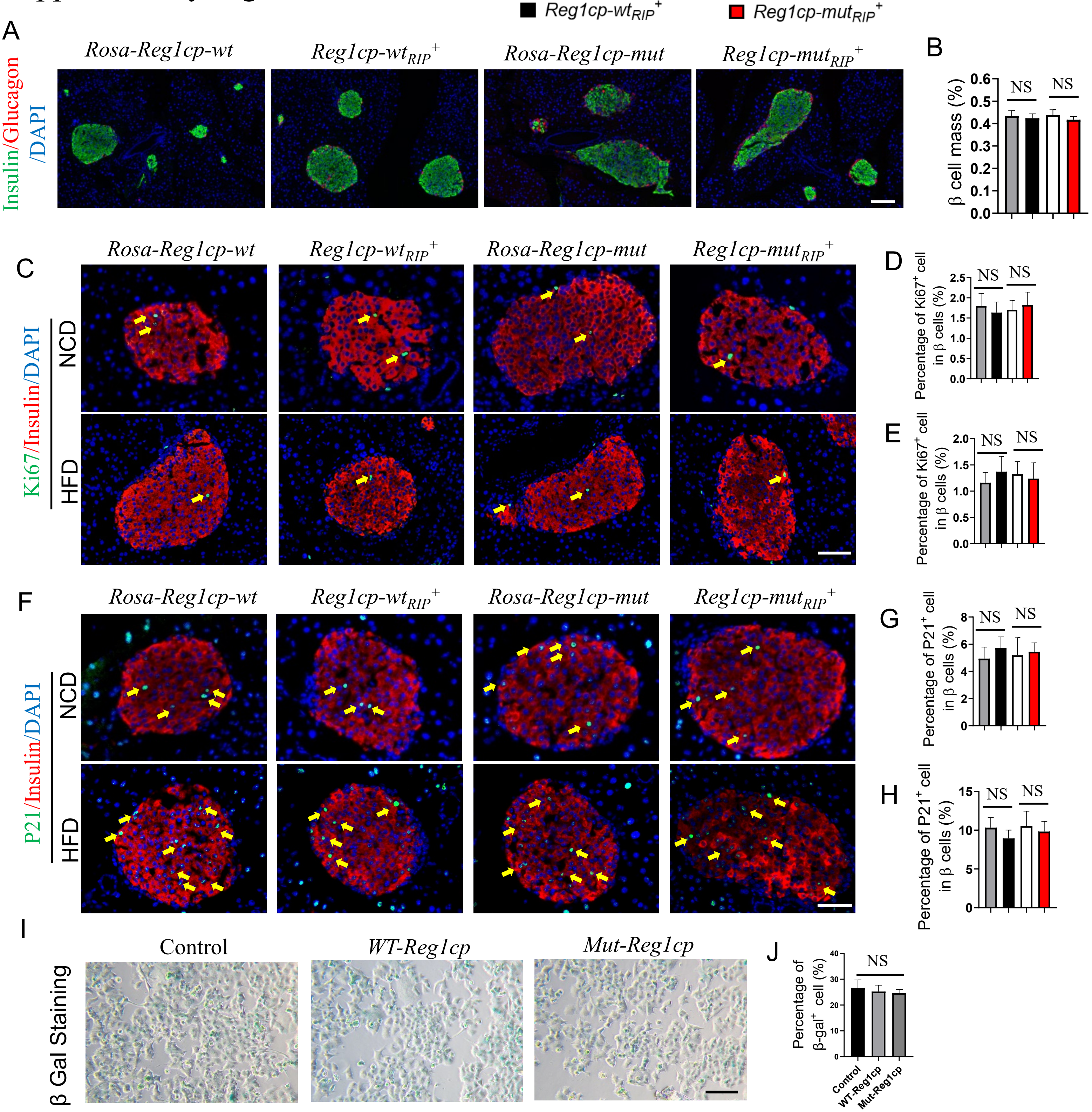

Supplementary Figure 2.

***WT-Reg1cp* or *Mut-Reg1cp* didn't affect the proliferation and senescence of β cells**

(A-B) Representative immunostaining images of insulin (green), glucagon (red) and DAPI, nucleus (blue) (A) and quantification of β cell mass (B) in *Reg1cp-wt*$_{RIP}$$^{+}$, *Reg1cp-mut*$_{RIP}$$^{+}$ mice and related *Rosa-Reg1cp-wt* and *Rosa-Reg1cp-mut* controls under HFD. Scale bar: 100μm. (C-E) Representative immunostaining image of Ki67 (green), insulin (red) and DAPI, nucleus (blue) (C) and quantification of Ki67 positive cells in *Reg1cp-wt*$_{RIP}$$^{+}$, *Reg1cp-mut*$_{RIP}$$^{+}$ mice and related *Rosa-Reg1cp-wt* and *Rosa-Reg1cp-mut* controls under normal chow diet (NCD) (D) or HFD (E). Scale bar: 50μm. (F-H) Representative immunostaining image of P21 (green), insulin (red) and DAPI, nucleus (blue) (F) and quantification of P21 positive cells in *Reg1cp-wt*$_{RIP}$$^{+}$, *Reg1cp-mut*$_{RIP}$$^{+}$ mice and related *Rosa-Reg1cp-wt* and *Rosa-Reg1cp-mut* controls under NCD (G) or HFD (H). Scale bar: 50μm. (I-J) Representative images of SA-βGal staining (I) and quantification of SA-βGal positive cells (J) in MIN6 cells transfected with Control, *Mut-Reg1cp* or *WT-Reg1cp* plasmids. Scale bar: 100μm. n = 6 in each group from three independent experiments. Data shown as mean ± SD. NS, no significance; One-way ANOVA.

Supplementary Figure 3

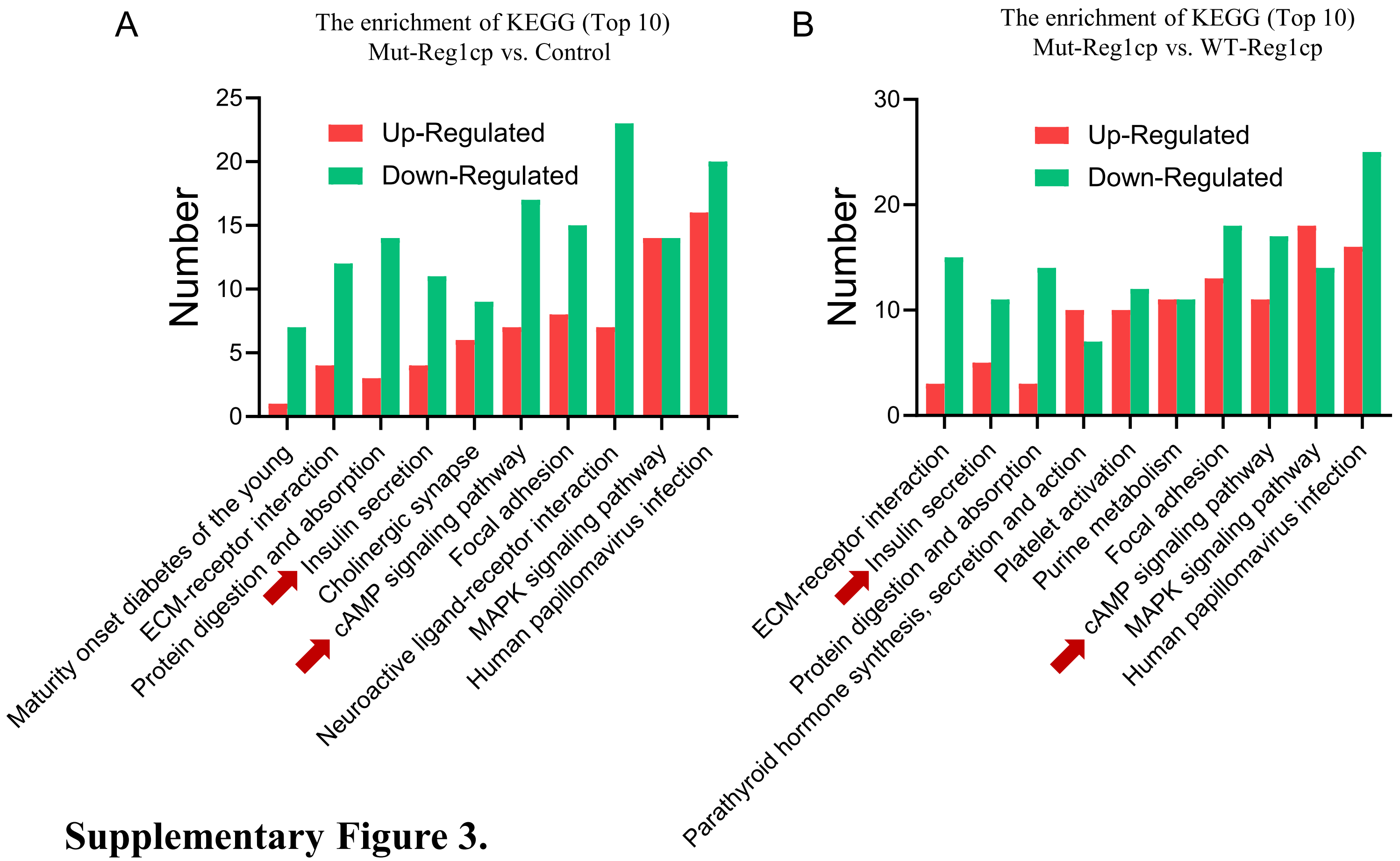

Supplementary Figure 3.

KEGG-enrichment analysis showed *Mut-Reg1cp* affected insulin secretion in MIN6 cells.

(A) The number of differentially expressed genes enriched in top 10 changed cellular functions indicated by KEGG-enrichment analysis of MIN6 cells transfected with *Mut-Reg1cp* or Control plasmids. n = 3. (B) The number of differentially expressed genes enriched in top 10 changed cellular functions indicated by KEGG-enrichment analysis of MIN6 cells transfected with *Mut-Reg1cp* or *WT-Reg1cp* plasmids. n = 3.

Supplementary Figure 4

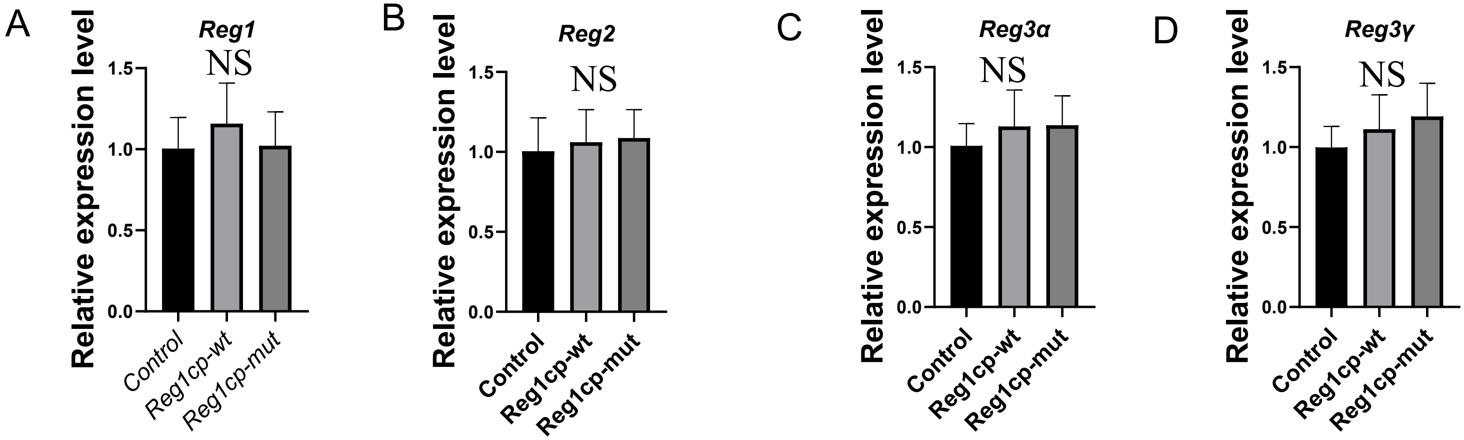

Supplementary Figure 4.

***Mut-Reg1cp* didn't affect the expression level of *Reg* family in MIN6 cells.**

(A-D) RT-PCR analysis of *Reg1* (A)*, Reg2* (B)*, Reg3α* (C) and *Reg3γ* (D) in MIN6 cells transfected with *WT- Reg1cp* or *Mut-Reg1cp* plasmids. n = 6 in each group from three independent experiments. Data shown as mean ± SD. NS, no significance; One-way ANOVA.

Supplementary Figure 5

A

Sequence	Mass	Leading razor protein	Intensity	Intensity_WT	Intensity_MUT
NNQFQALLQYADPVSAQHAK	2242.1131	Q3TQW3	4466600	0	4466600
VTPQSLFILFGVYGDVQR	2038.0888	Q3TQW3	3624300	0	3624300
KLPSDVTEGEVISLGLPFGK	2085.1358	Q3TQW3	2556800	0	2556800

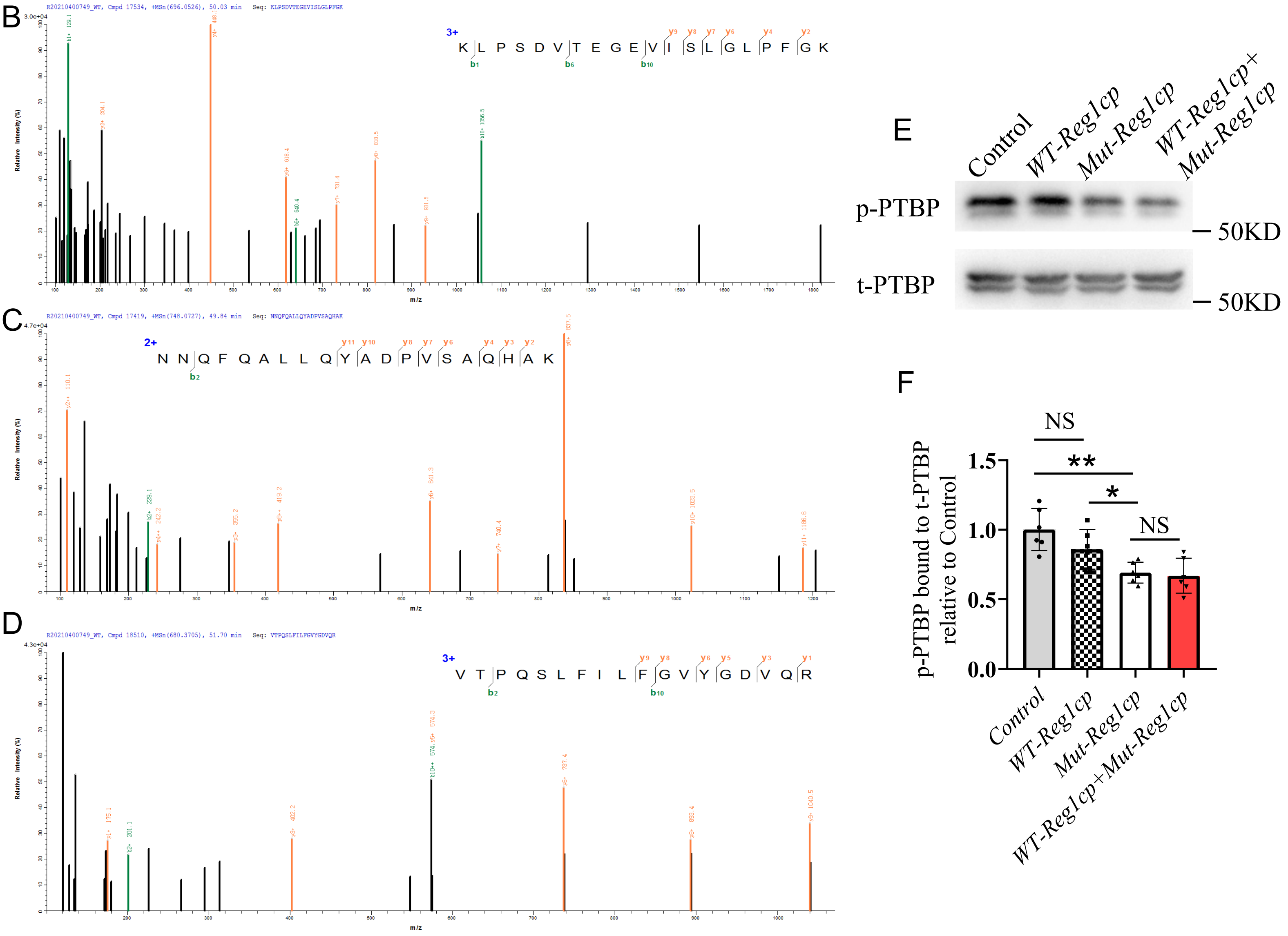

Supplementary Figure 5.

Peptides belongs to PTBP1 retrieved by *Mut-Reg1cp*.

(A-D)Information of three peptides belong to PTBP1 retrieved by *Mut-Reg1cp* which was identified by MS. (E-F) Representative pictures (E) and quantitative measurements (F) of western blot analysis of the expression of phosphorylation and total PTBP1 (p-PTBP1 and t-PTBP1) in MIN6 cells transfected Control, *WT- Reg1cp, Mut-Reg1cp* or *WT- Reg1cp*+*Mut-Reg1cp* plasmids. Data shown as mean ± SD. *, $P < 0.05$; **, $P < 0.01$; NS, no significance; One-way ANOVA.

Supplementary Figure 6

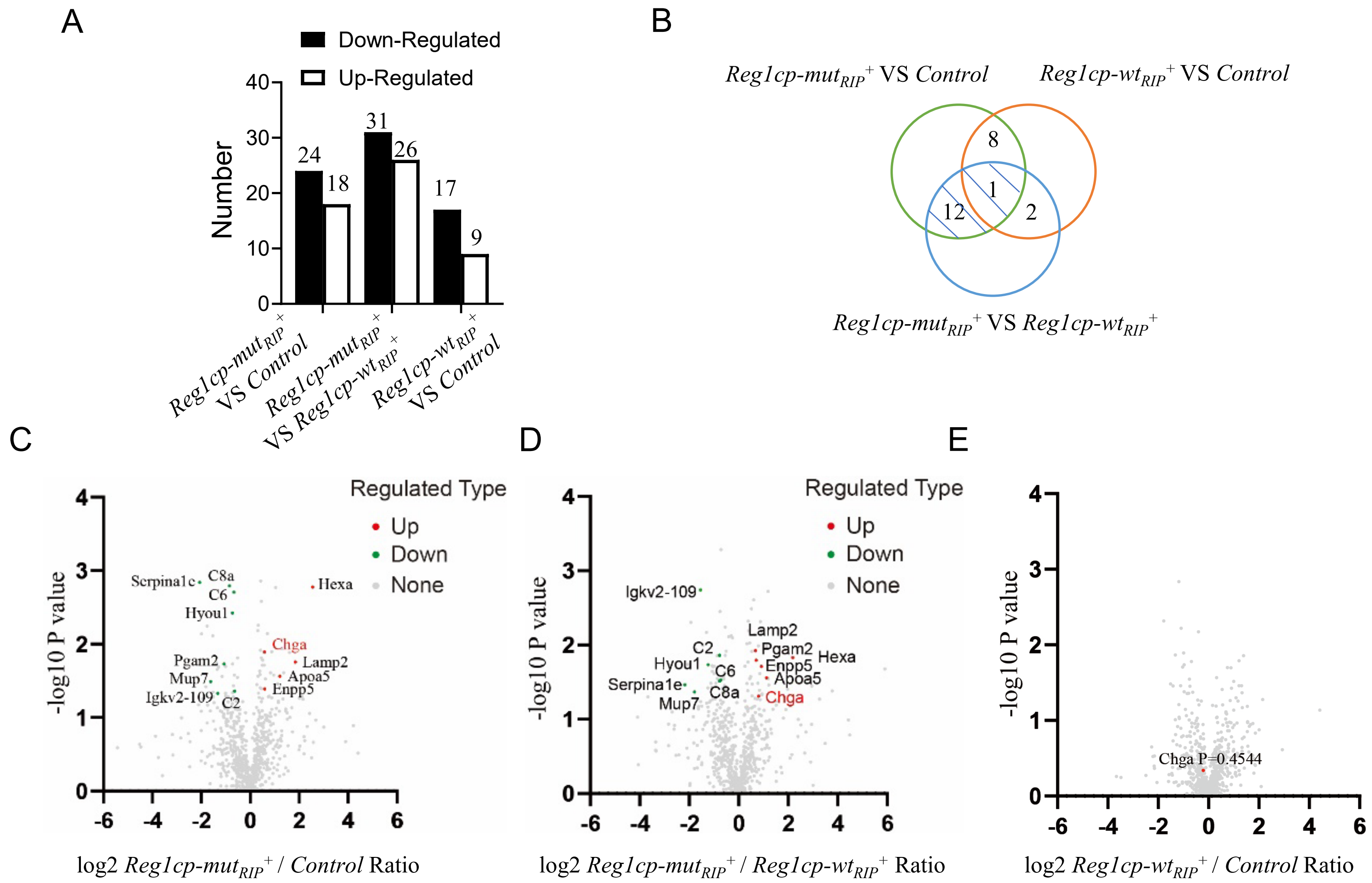

Supplementary Figure 6. Circulating proteins were not the cause of insulin resistance in *Reg1cp-mut*$_{RIP}^{+}$ mice.

(A) Bar graph showed serum proteins whose level showed significant differences between control and *Reg1cp-mut*$_{RIP}^{+}$ mice, *Reg1cp-wt*$_{RIP}^{+}$ and *Reg1cp-mut*$_{RIP}^{+}$ mice or *Reg1cp-wt*$_{RIP}^{+}$ and control mice. (B) Venn diagram showed overlapping of serum proteins whose level showed significant differences between control and *Reg1cp-mut*$_{RIP}^{+}$ mice, *Reg1cp-wt*$_{RIP}^{+}$ and *Reg1cp-mut*$_{RIP}^{+}$ mice or *Reg1cp-wt*$_{RIP}^{+}$ and control mice. (C-E) Volcano plot showed serum proteins whose level showed significant differences between control and *Reg1cp-mut*$_{RIP}^{+}$ mice (C) *Reg1cp-wt*$_{RIP}^{+}$ and *Reg1cp-mut*$_{RIP}^{+}$ mice (D) or *Reg1cp-wt*$_{RIP}^{+}$ and control mice (E).
