# Supplementary material for: Mutated lncRNA increase the risk of type 2 diabetes by promoting β cell dysfunction and insulin resistance
Source: Cell Death Dis. 2022 Oct 27;13(10):904. doi: 10.1038/s41419-022-05348-w (PMC9613878; doi:10.1038/s41419-022-05348-w)
Supplement: Supplementary file 2 — Supplementary Table 1 [file 41419_2022_5348_MOESM2_ESM.docx]

**Supplementary Table 1. Peptides only be retrieved by Mut-Reg1cp or WT-Reg1cp by Mass spectrometry analyze after RNA pulldown.**

| Sequence | Mass | Leading razor protein | Charges | Intensity | Intensity _WT | Intensity _MUT |
| --- | --- | --- | --- | --- | --- | --- |
| INHEGEVNRARYMPQNPHIIATK | 2687.371 | F6ZLC6 | 2 | 75796000 | 75796000 | / |
| DTKCTVTPLTEGSLYVFRVAAENAIGQSDYTEIGDSVLAK | 4318.132 | A0A5K1VVQ1 | 5 | 62303000 | 62303000 | / |
| MISHIEDVESK | 1286.618 | Q9CUR1 | 1 | 20952000 | 20952000 | / |
| MHQGQLEIDK | 1197.581 | G5E881 | 2 | 1352300 | 1352300 | / |
| AKFNIGFGDR | 1123.577 | Q5NCP6 | 1 | 503550000 | / | 503550000 |
| LQQSLQQGNHSSGSNR | 1739.83 | A0JLT5 | 3 | 10838000 | / | 10838000 |
| SEFKDEPLLFRFFADEEMEGSNMK | 2895.31 | Q3LAC4 | 4 | 4939500 | / | 4939500 |
| PSITNSGGHR | 1024.505 | D3YXG0 | 1 | 4664800 | / | 4664800 |
| NNQFQALLQYADPVSAQHAK | 2242.113 | Q3TQW3 | 3 | 4466600 | / | 4466600 |
| VTFLEEVTEYYISGDEDRK | 2292.08 | Q8BFW3 | 4 | 4353400 | / | 4353400 |
| ALDLIEVLVTK | 1212.733 | A0JLT5 | 2 | 4222100 | / | 4222100 |
| DGDGVPGGR | 828.3726 | Q6KAM6 | 2 | 3693100 | / | 3693100 |
| VTPQSLFILFGVYGDVQR | 2038.089 | Q3TQW3 | 3 | 3624300 | / | 3624300 |
| KLPSDVTEGEVISLGLPFGK | 2085.136 | Q3TQW3 | 3 | 2556800 | / | 2556800 |
| FDMQVTVPR | 1091.543 | O88967 | 2 | 2420700 | / | 2420700 |
| LLAGDHPIELLLRDFKK | 1977.141 | Q3UYK7 | 6 | 1700800 | / | 1700800 |
| AAVAGEDGR | 844.4039 | A0A2I3BQH3 | 2 | 1663400 | / | 1663400 |
| QAVVTQESALTTSPGETVTLTCR | 2448.217 | A2MXS5 | 3 | 781810 | / | 781810 |
| YCHEVGPCAEALHAQVER | 2124.947 | A0JLT5 | 4 | 725290 | / | 725290 |
| VHWTFKFFDSQGARSR | 1967.976 | Q8BRB3 | 6 | 258500 | / | 258500 |
