# Supplementary material for: Mutated lncRNA increase the risk of type 2 diabetes by promoting β cell dysfunction and insulin resistance
Source: Cell Death Dis. 2022 Oct 27;13(10):904. doi: 10.1038/s41419-022-05348-w (PMC9613878; doi:10.1038/s41419-022-05348-w)

Figure 4 A

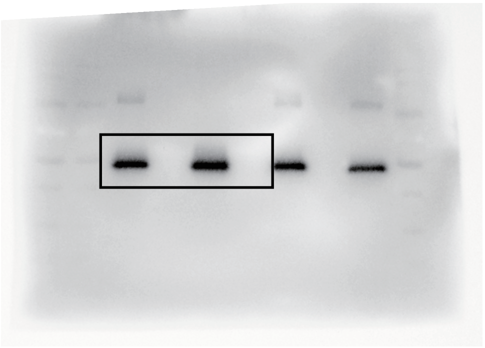

Figure 4 C

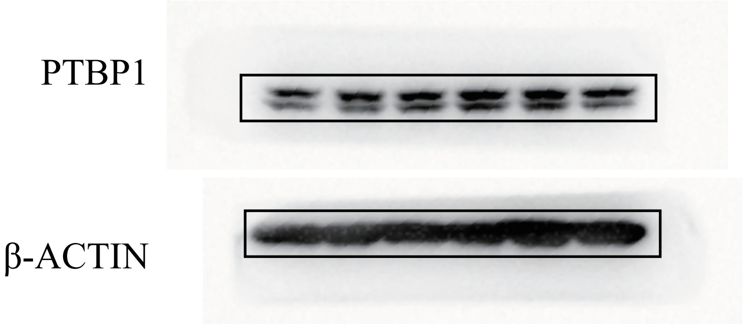

Figure 4 E

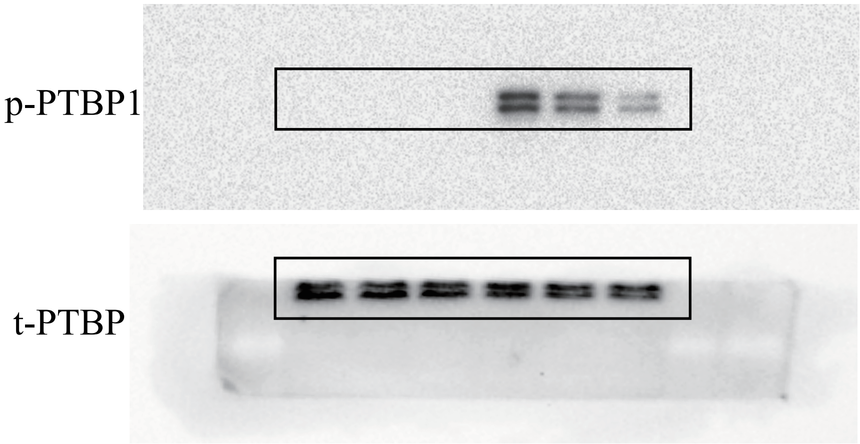

Figure 4 I

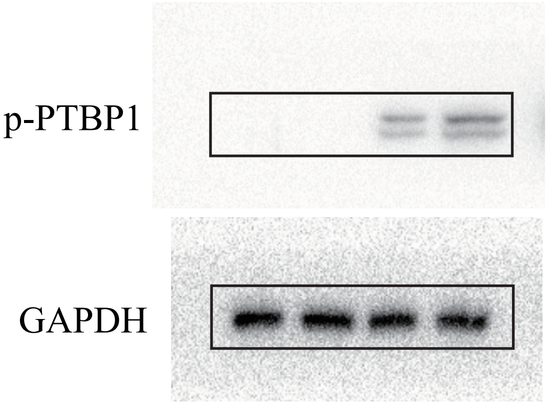

Figure 4 F

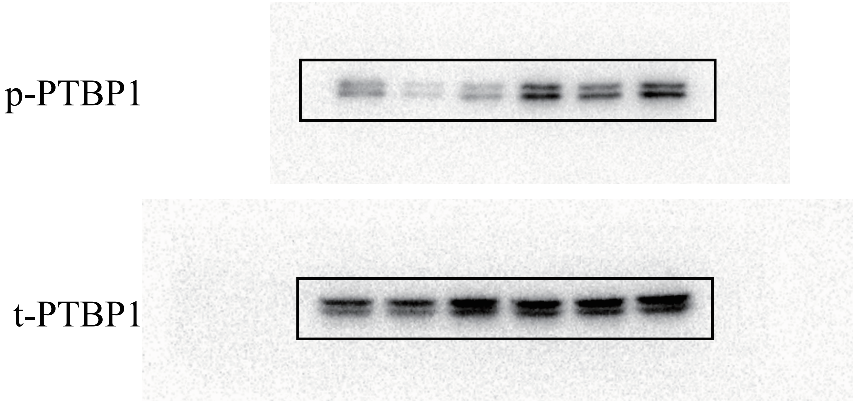

Figure 6 H Figure 5 A

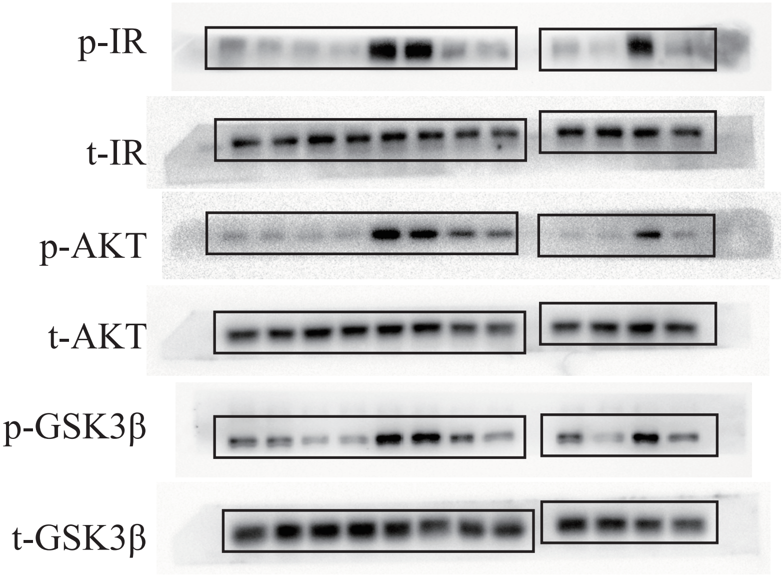

Figure 6 J Figure 5 C

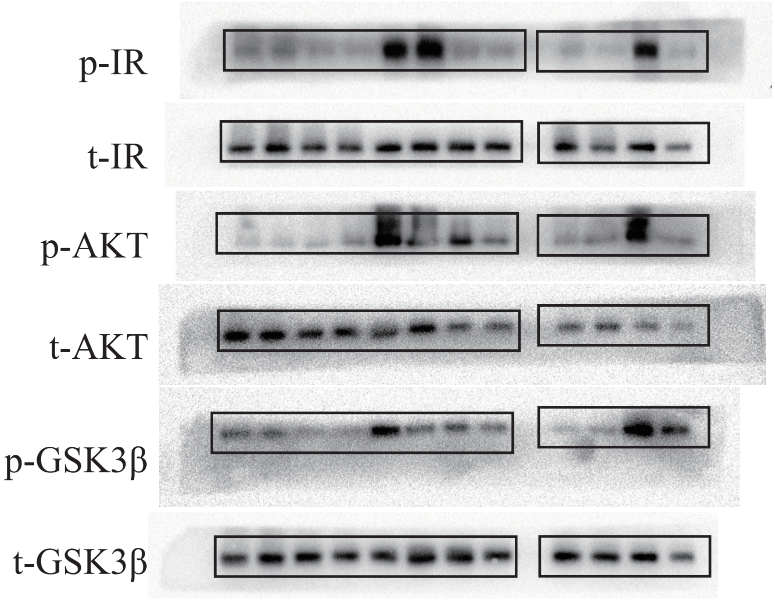

Figrue 5 E

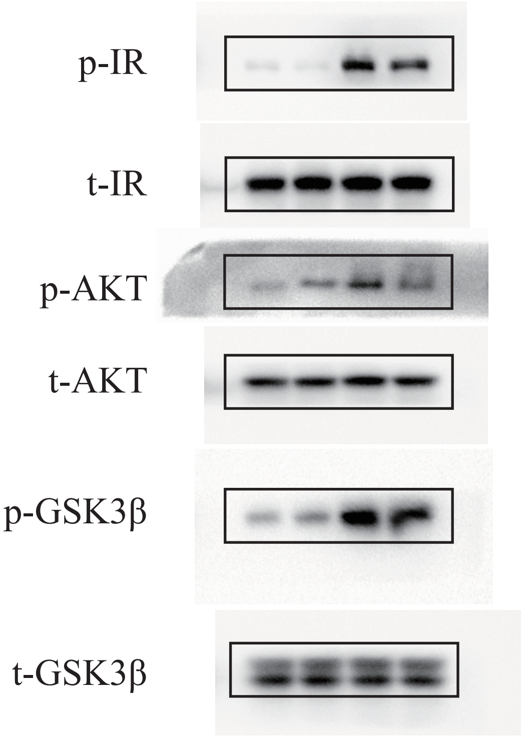

Figure 5 I

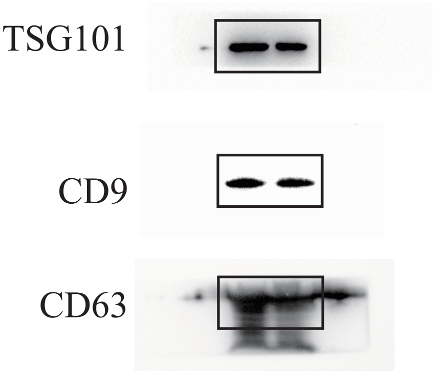

Figure 5 H

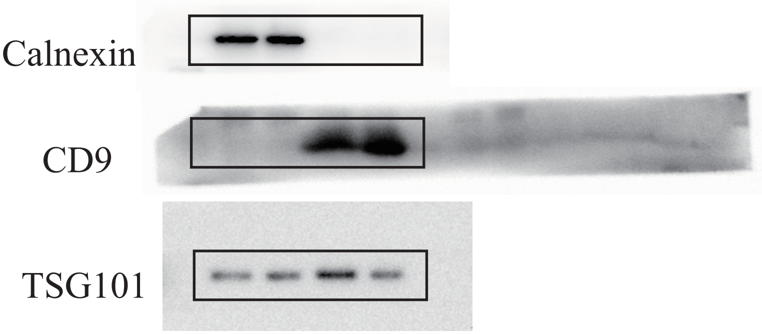

Figure 7 A

PTBP1

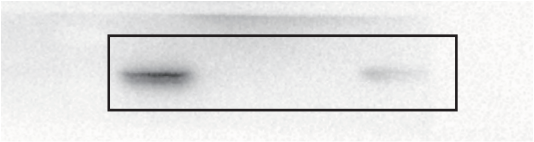

Figure 7 B

PTBP1
ADIPOR1
TUBLIN

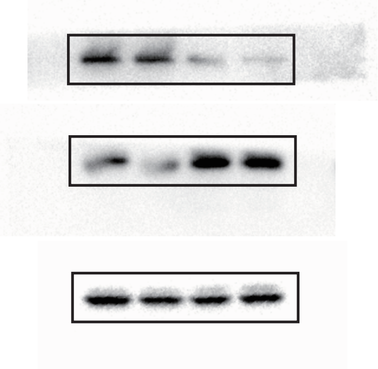

Figure 7 D

ADIPOR1
TUBLIN

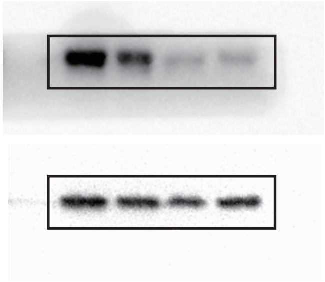

Figure 7 F

ADIPOR1
TUBLIN

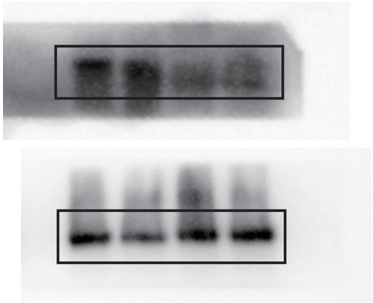

Supplementary Figure 5 E

p-PTBP1
t-PTBP1

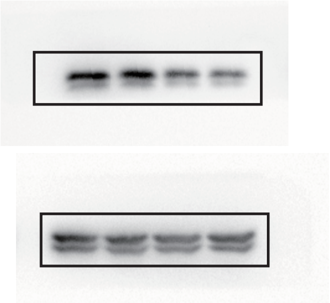

Figure 7 H

ADIPOR1
TUBLIN

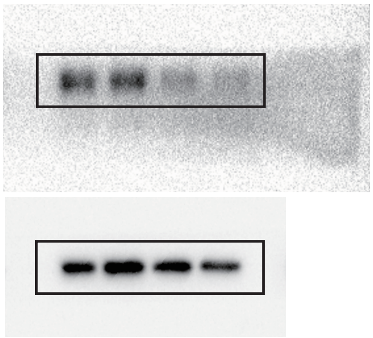

Figure 7 J

ADIPOR1
TUBLIN

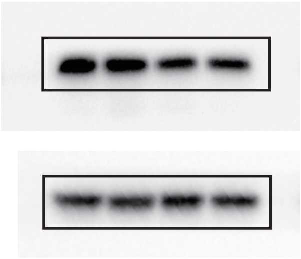

Figure 7 M

ADIPOR1
P-AMPK
t-AMPK
TUBLIN

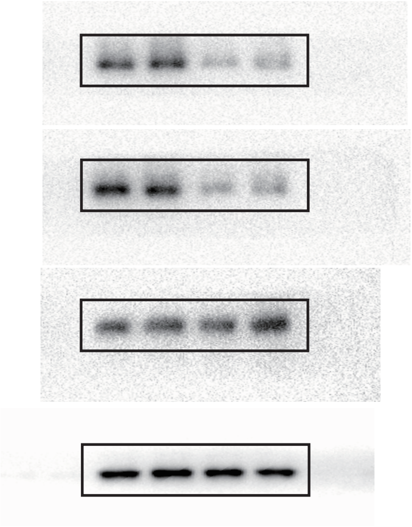

Figure 4 B

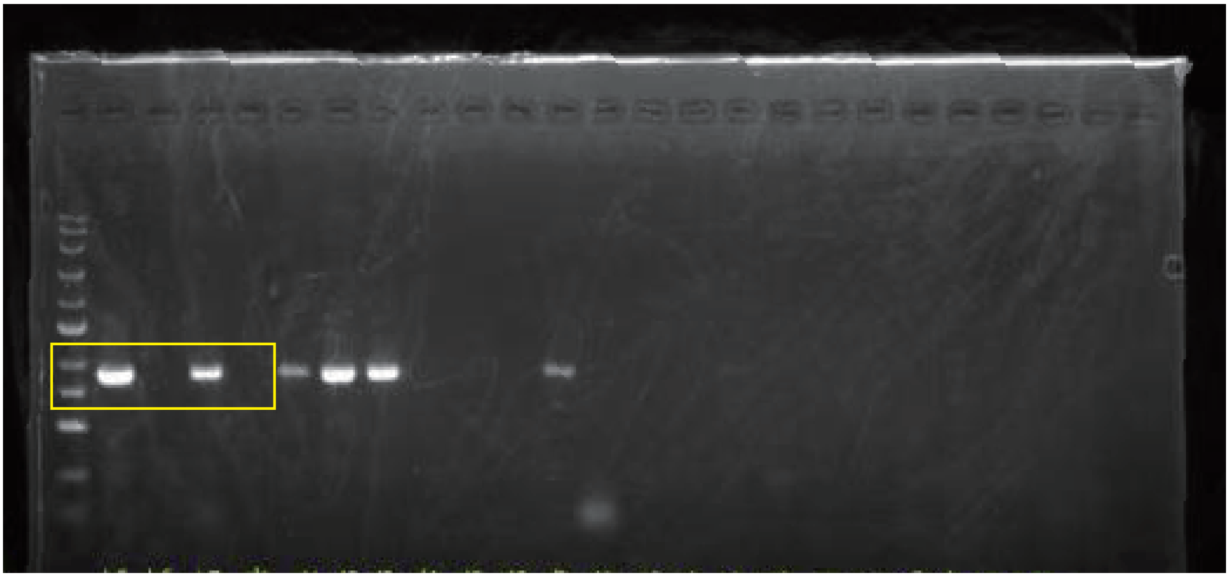

Figure 5 G

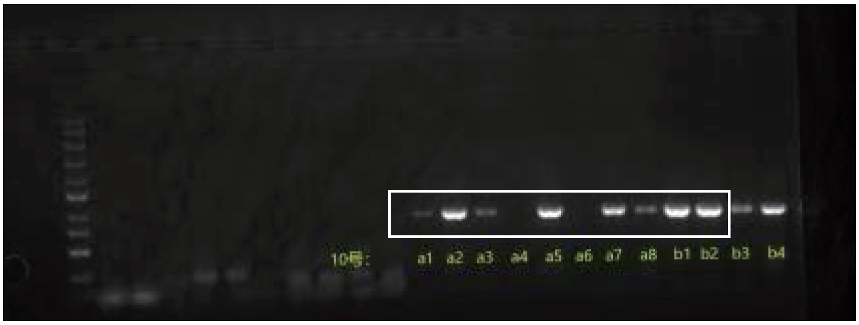

Figure 5 J

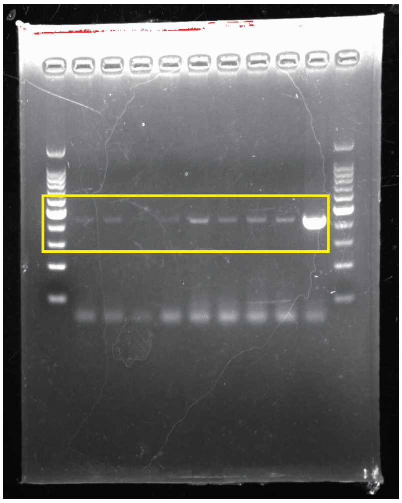

Figure 7 L

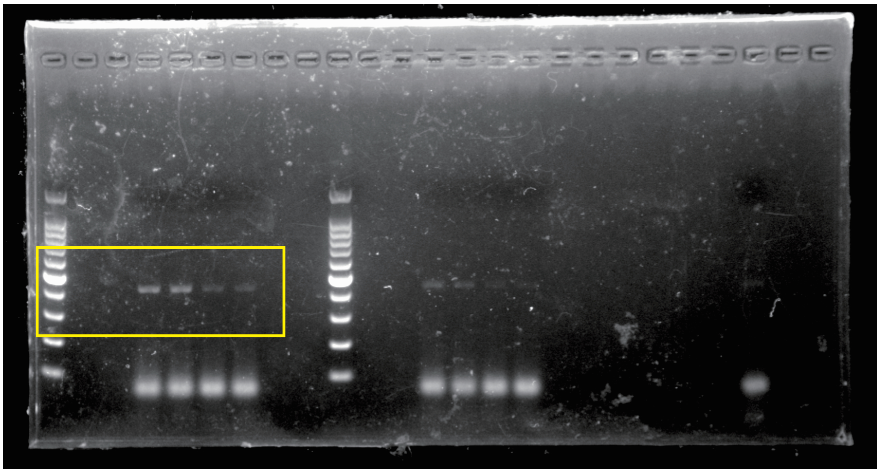

Supplement: Supplementary file 3 — Full uncut gels [file 41419_2022_5348_MOESM3_ESM.pdf]
